# Quantitative visualization of myocardial ischemia-reperfusion-induced cardiac lesions via ferroptosis magnetic particle imaging

**DOI:** 10.7150/thno.89190

**Published:** 2024-01-01

**Authors:** Wenwen Yang, Yueqi Wang, Changgeng Fu, Changjian Li, Feng Feng, Hongzheng Li, Ling Tan, Hua Qu, Hui Hui, Jingjing Wang, Jie Tian, Linzi Long

**Affiliations:** 1 Department of Cardiology, Xiyuan Hospital of China Academy of Chinese Medical Sciences, Beijing, 100091, People's Republic of China.; 2 National Clinical Research Center for Cardiovascular Diseases of Traditional Chinese Medicine, Beijing, 100091, People's Republic of China.; 3 CAS Key Laboratory of Molecular Imaging, Beijing Key Laboratory of Molecular Imaging, the State Key Laboratory of Management and Control for Complex Systems, Institute of Automation, Chinese Academy of Sciences, Beijing, 100190, People's Republic of China.; 4 School of Engineering Medicine, Beihang University, Beijing 100191, People's Republic of China.; 5 College of Energy Engineering, Zhejiang university, Zhejiang 310058, People's Republic of China.; 6 Beijing University of Traditional Chinese Medicine Graduate School, Beijing University of Chinese Medicine, Beijing, 100105, People's Republic of China.; 7 Department of Cardiovascular Medicine, First Medical Center, General Hospital of the People's Liberation Army of China,Beijing, 100853, People's Republic of China.; 8 Academy of Integrative Medicine, Fujian University of Traditional Chinese Medicine, Fuzhou, 350122, People's Republic of China.

**Keywords:** myocardial ischemia-reperfusion, ferroptosis, magnetic particle imaging, quantitative, visualization

## Abstract

Myocardial ischemia-reperfusion (MI/R) injury is a complication in vascular reperfusion therapy for MI, occurring in approximately 60% of patients. Ferroptosis is an important process in the development of MI/R cardiac lesions. Transferrin receptor 1 (TfR1), a marker of ferroptosis, corresponds to the changes in MI/R cardiac lesions and is expected to be a biomarker for detecting MI/R-induced ferroptosis. However, the noninvasive *in vivo* visualization of ferroptosis in MI/R is a big challenge. Thus, this study aimed to develop a novel multimodal imaging platform to identify markers of MI/R cardiac lesions *in vivo* through targeting TfR1.

**Methods:** Magnetic particle imaging (MPI) modality for ferroptosis based on superparamagnetic cubic-iron oxide nanoparticles (SCIO NPs), named feMPI, has been developed. FeMPI used TfR1 as a typical biomarker. The feMPI probe (SCIO-ICG-CRT-CPPs NPs, CCI NPs) consists of SCIO NPs, TfR1-targeting peptides (CRT), cell-penetrating peptides (CPPs), and indocyanine green (ICG). The specificity and sensitivity of CCI NPs in the MI/R mouse model were evaluated by MPI, magnetic resonance imaging (MRI), and near-infrared (NIR) fluorescent imaging.

**Results:** The intensity of the MPI signal correlates linearly with the percentage of infarct area in MI/R stained by TTC, enabling a quantitative assessment of the extent of cardiac lesions. Notably, these findings are consistent with the standard clinical biochemical indicators in MI/R within the first 24 h. FeMPI detects cardiac injury approximately 48 h prior to the current clinical imaging detection methods of MI/R.

**Conclusion:** The feMPI strategy can be a powerful tool for studying the process of MI/R-induced ferroptosis *in vivo*, providing clues for molecular imaging and drug development of ferroptosis-related treatments.

## Introduction

The most effective therapy for limiting infarct severity and improving clinical outcomes following acute myocardial infarction (MI) is the rapid restoration of blood flow through the occluded coronary artery via primary percutaneous coronary intervention 1. Compared with thrombolytic therapy, primary percutaneous coronary intervention exhibits reduced morbidity and mortality; however, myocardial ischemia-reperfusion (MI/R) lesions, a complication of vascular reperfusion therapy for acute myocardial infarction, can occur in ≥ 60% of patients 2. Specifically, reperfusion injury may be independently associated with adverse left ventricular (LV) remodeling, an increased risk of fatal arrhythmias, and hospitalization for heart failure 3.

Ferroptosis, an iron-dependent form of regulated cell death, has been increasingly recognized as an important process that mediates the pathogenesis and progression of numerous cardiovascular diseases, including MI/R injury, sepsis-induced cardiomyopathy, and arrhythmia 4,5. Apoptosis and necrosis were observed in the early stage of I/R injury. However, during prolonged reperfusion, we found ferroptosis to be the predominant form of cell death 5,6. Early in ferroptosis, there is the enhancement of iron uptake, which results in the high expression of transferrin receptor 1 (TfR1). TfR1 is a cell-surface protein that binds to transferrin in the blood to facilitate iron uptake 7. This altered expression of TfR1 and the modified transport of iron from the cytoplasm to mitochondria lead to an accumulation of iron in the mitochondria. Additionally, these changes induce the endocytosis of reticulosin-dependent complexes 8,9. Hence, TfR1 is a promising biomarker for specifically detecting MI/R-induced ferroptosis 10. However, existing detection methods are insufficient for noninvasive and sensitive detection of abnormal TfR1 expression in the early stages of injury, and novel technical approaches are urgently needed to resolve this limitation. Molecular imaging has become an important tool in the diagnosis and treatment of MI/R, which can detect the disease at molecular and cellular levels 11. Currently, imaging modalities for identifying cardiac injury in clinical practice are restricted when used for the quantitative detection of TfR1; thus, advanced molecular imaging techniques have been devoted to visualizing MI/R changes, such as positron emission tomography (PET) and magnetic resonance imaging (MRI) 12,13,14. Moreover, near-infrared (NIR) fluorescent molecular imaging has also been used for viewing cardiac injury in small animal models; however, the depth of the image is reduced due to light absorption and scattering 15,16. Comparatively, when used for cardiac imaging, the motion effects closely related to the cardiac cycle and diaphragm motion have limited MRI image efficiency 17. Furthermore, MRI and PET acquisition times are relatively long. It is necessary to develop imaging strategies that can detect ferroptosis and TfR1 *in vivo* with high sensitivity for the detection of MI/R-induced cardiac injury.

Magnetic particle imaging (MPI) is a new tomographic technique that detects changes in the electronic magnetization of iron. MRI, on the other hand, measures changes in the nuclear magnetization of water protons 18. Therefore, MPI is much more sensitive than MRI, with less background signal noise and negligible attenuation of tissues. In addition, MPI has the advantage of detecting the number of superparamagnetic iron oxide nanoparticles (SPIONs) and producing a positive signal. MPI allows non-invasive quantitative monitoring of marker biodistribution without the use of ionizing radiation. Therefore, it is very suitable for the quantitative detection of TfR1 changes *in vivo*. The combination of MPI, NIR, and MRI complements each other because MPI has a relatively low spatial resolution and does not provide anatomical information. To provide deeper and more accurate anatomical details for this, it is necessary to develop MPI of ferroptosis (feMPI) and corresponding NIR and MRI contrast agents. Therefore, different imaging approaches combining multimodal imaging markers are needed to detect MI/R injury. Optical imaging methods, such as near-infrared fluorescent, with a high sensitivity and spatial resolution allow real-time monitoring of nanoparticle pharmacokinetics and biodistribution 19. MRI can provide high-resolution deep tissue morphology and anatomical details and is generally used clinically. When using contrast agents, the sensitivity of MRI is limited 20. Therefore, developing multimodal imaging techniques that integrate the complementary advantages of different modalities is imperative 21,22,23.

This study aimed to design a probe that is easily absorbed by cells to actively target TfR1. Superparamagnetic cubic-iron oxide nanoparticles (SCIO NPs), indocyanine green (ICG), TfR1-targeting peptides (CRT) 24, and cell-penetrating peptides (CPPs) 25,26 were the main components of the probe. Among these, SCIO NPs possess MPI/MRI imaging properties, ICG has optical imaging properties, CRT can interact with TfR1 through nonclassical ligand-guided mechanisms, and NPs can accumulate in MI/R tissues 27. CPPs are considered promoters of intracellular delivery and have developed binding targeting specificities in recent years 28; thus, they express great potential for the targeted delivery of probes. Accordingly, CPPs were utilized here to promote the entry of nanoparticles into cells, thereby overcoming the problem of low uptake efficiency in myocardial cells 29,30.

**Scheme 1:**
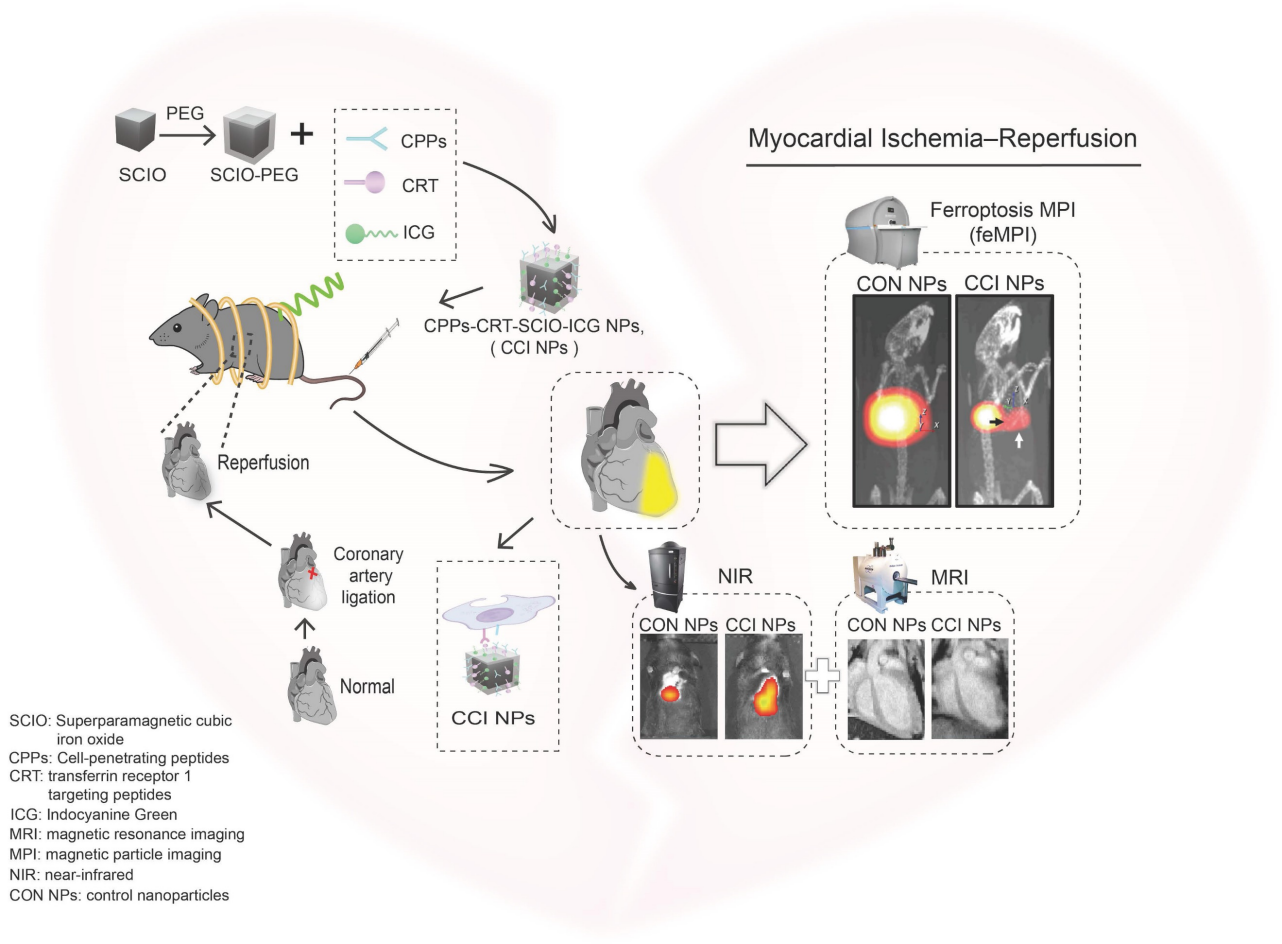
Schematic of feMPI and NIR/MRI multimodal imaging for MI/R using the targeting contrast agent CCI NPs.

Targeting peptides and ICG were directly conjugated to SCIO NPs using simple chemistry, taking advantage of the high sensitivity and specificity of feMPI. Additionally, multimodal imaging combined with NIR/MRI further detected MI/R-induced cardiac injury (Scheme 1). It was found that SCIO-ICG-CRT-CPPs NPs (CCI NPs) showed an even denser distribution and longer retention in MI/R-injured tissue than those of SCIO-ICG NPs (CON NPs) which were absorbed easily by the liver where the signal was concentrated. The results of feMPI were consistent with those of cardiac troponin I (cTnI) in the first 24 h, which is a commonly used clinical indicator in MI/R, with dual-targeted probes identifying MI/R-induced cardiac injury ≥ 48 h earlier than echocardiography. Overall, the CCI NPs multimodal imaging probe has high MI/R detection sensitivity, excellent targeting ability, and high reliability. To the best of our knowledge, this is the first report of *in vivo* MI/R imaging with feMPI and integrated NIR/MRI has a more complete MI/R characterization and a higher detection accuracy.

## Materials and Methods

### Synthesis of SCIO-PEG NPs

Iron oxide cores were coated with oleic acid (OA; Aladdin Co., Ltd., Shanghai, China) to synthesize SCIO-OA NPs using the thermal decomposition method, as previously described 37. The idiographic processes were as follows: iron (III) acetylacetonate (0.84 g, Aladdin), oleylamine (2.04 mL, OAm; Aladdin Co., Ltd., Shanghai, China), OA (2.76 mL), and dibenzyl ether (24 mL; Aladdin Co., Ltd., Shanghai, China) were added to a 60-mL three-neck flask. Then, nitrogen (N_2_) flow was stirred in the mixture as it was heated to nucleation temperature (220 °C) at a rate of 3.3 °C/min for 1 h. The mixture was then heated to 300 °C and maintained for 12 h. The product was sonicated and purified three times, where a strong magnet was used to remove residual OAm, OA, and dibenzyl ether. The product was then dispersed in chloroform (10 mL, Aladdin Co., Ltd., Shanghai, China). The surface of SCIO-OA was modified using 1,2-Distearoyl-sn-glycero-3-phosphoethanolamine2000-N-amino (DSPE-PEG2000-NH_2;_ Nanjing Nanoeast Biotech Co., Ltd., Nanjing, China). The citric-coated SCIO particles were diluted to 1 mg/mL in deionized water. The pH of the mixture was adjusted to 11 using an ammonia solution. A total of 10 mg DSPE-PEG2000-NH_2_ was modified to the SCIO-OA NPs and reacted for 1 d with agitation. The reaction system was heated to 80 °C for 2 h for the covalent linking between SCIO NPs and DSPE-PEG2000-NH_2_. The resultant SCIO-DSPE-PEG-NH_2_NPs (SCIO-PEG NPs) were concentrated with ThermoFisher Heraeus Fresco 21 centrifugal filters and stored at 4 °C.

### Synthesis of CCI/CRT/CPPs/CON NPs

CCI NPs were then synthesized using the following procedure: functionalization of SCIO NPs with TfR1-targeting peptides, CRT (CRTIGPSVC; GL Biochem Co., Ltd., Shanghai, China), and CPPs (RKKRRQRRR, GL Biochem Co., Ltd.) was accomplished using a sulfo-SMCC (InnoChem, Co., Ltd., Beijing, China) crosslinker. Among them, SMCC contains two terminal functional groups: the sulfhydryl-reactive maleimide group, and the amine-reactive NHS ester group. For the available amine groups, 2-molar excess sulfo-SMCC was used and reacted with the SCIO-NH_2_ NPs for 30 min. Then, a 4:3:3 molar excess of sulfo-SMCC was added to the (Mpa)-CRT and (Mpa)-CPPs and then reacted for another 3 h with shaking. The obtained mixture containing CRT-CPPs-SCIO NPs (CC NPs) was dialyzed with a 1 × 10^5^ Da molecular weight cut-off dialysis bag to remove the redundant targeting peptides and sulfo-SMCC. After dissolving the obtained CC NPs in deionized water with a pH of 8.5, we added ICG-N-hydroxysuccinimide (ICG-NHS) ester (Solarbio Co., Ltd., Beijing, China), stirred it at 20 °C, and reacted it with the CC NPs solution overnight to achieve CCI NPs. The mixed solution was centrifuged (5900 × g) for 0.5 h to remove the excess ICG-NHS ester. SCIO-ICG-CRT NPs (CRT NPs) were synthesized by conjugating the terminal amine groups of the composite SCIO-NH2 NPs with the thiol groups on the serine end of the peptides, for which (Mpa)-CRT (a sulfhydryl modified TfR1-targeting peptides) and ICG-NHS were used. SCIO-ICG-CPPs NPs (CPPs NPs) were synthesized by conjugating the terminal amine groups of the composite SCIO-NH2 NPs with the thiol groups on the serine end of the peptides, for which (Mpa)-CPPs (a sulfhydryl modified cell-penetrating peptides) and ICG-NHS were used. CON NPs were synthesized by conjugating ICG-NHS ester to SCIO-PEG NPs. ICG-NHS was mixed into the SCIO-PEG NPs solution by adjusting to 8.5 at a 1:5 ratio of amine-to-dye groups and stirred for 12 h at 20 °C. The mixed solution was centrifuged at (5900 × g) for 0.5 h to remove the excess ICG-NHS. All the above reactions were performed in a dark environment.

### Characterization of CCI NPs

When preparing for detection, CCI NPs were dissolved in deionized water. The SCIO core size and morphology of CCI NPs were detected using TEM (JEM-2100F, JEOL, Tokyo, Japan). Measuring the dynamic magnetization curve of CCI NPs and Vivo Trax used magnetic particle spectroscopy (MPS) under measurement conditions of 1kHz, 20mT/\ mu_0. The FTIR spectra of PEG, ICG-NHS, CPPs, SCIO-PEG-NH_2_, CON NPs, and CCI NPs were obtained with the FTIR spectrometer (Thermo-Electron, Nicolet IS10, USA). The zeta potential and hydrodynamic diameters of CON\CRT\CCI NPs were measured with Zetasizer Nano ZS (Malvern, UK) at 25 °C. The fluorescent spectro photometer F-7000 (Hitachi, Tokyo, Japan) was used to measure the emission spectra of ICG-NHS ester and CCI NPs at 25 °C. The optical absorption analysis of CCI and CON NPs was detected using a UV-Vis-NIR spectrophotometer (Shimadzu, Japan). The MPI signal, fluorescent intensity, and MRI signal analysis of CCI NPs were obtained at different concentrations with a Momentum MPI scanner (Magnetic Insight Co., USA), the IVIS spectrophotometer (PerkinElmer, USA), and 7 T preclinical MRI system (BioSpec 70/30 USR, Bruker BioSpin, Billerica, MA, DE).

### Cell culture and establishment of I/R cell models

When the degree of fusion of H9c2 in myocardial cells was 80%, the model was established, and the cells were washed twice with PBS. Oxygen-glucose deprivation and reoxygenation were then used to simulate I/R. First, oxygen-glucose deprivation was performed, and the cell culture medium was replaced with a sugar-free medium. The culture was then placed in a 95% N_2_ and 5% CO2 hypoxic incubator for 6 h. The medium was then replaced with a normal, sugar-containing cell culture medium, and cultured in a 95% N_2_ and 5% CO_2_ incubator for 16 h.

### Experimental grouping and processing

For the blank control group, inoculated cells were placed into a dedicated culture medium that was replaced after 24 h with a culture medium containing 10% blank serum added for routine cultivation. For the model control group, cells were inoculated into a dedicated culture medium that was removed after 24 h. Following stimulation by hypoxia and reoxygenation, a culture medium containing 10% blank serum was added, and the cells were treated for 24 h.

### CCK8 testing for cytotoxicity

H9c2 cells were cultured in dulbecco's modified eagle medium (DMEM) containing 10% fetal bovine serum (FBS). H9c2 cells were cultured at a density of 2 × 10^4^ cells/well in 96-well plates and treated for 24 h at 37 °C, 5% CO_2_ (Heracell, Thermo Fisher Scientific). In this study, the I/R H9c2 cell model was established as described above. To test the effects of CON and CCI NPs, the probes were diluted in the DMEM solution containing serum. The Fe concentration used ranged from 0 to 80 µg/mL, with increments of 10 µg/mL. Next, NPs and a control medium were used. Following 24 h, cells were washed three times with PBS, and 10 µL of CCK-8 was added before incubating in DMEM for 2 h. The optical density (OD) values were detected at 450 nm, and these OD values were analyzed as the percentage of control pore value with a multimodal microplate reader (Bio Tek, USA).

### Ethics

All animal studies were performed according to the guidelines of the Institutional Animal Care and Use Committee at Xiyuan Hospital China Academy of Chinese Medical Sciences (No.2021XLC027).

### Establishment of MI/R mouse model

Seven-week-old C57BL/6N mice were purchased from Beijing Charles River Experimental Animal Technology Company, China. In the MI/R mouse model, 2% isoflurane was used to anesthetize the mice. Following this, the skin was cut between the third and fourth ribs on the left side of the mouse and the muscles were passively separated layer-by-layer, quickly extruding the myocardium and ligating the anterior descending branch of the coronary artery of the heart, approximately 2 mm below the junction of the left atrial appendage and conus arteriosus. The heart was quickly placed back into the heart cavity and the skin was sutured. After 30 min of ligation, the ligature thread was removed to restore cardiac perfusion. When the electrocardiogram showed obvious ischemia, the MI/R mice were randomly divided into two groups, as follows: (1) CON NPs and (2) CCI NPs. Each group was injected with 100 µL of nanoparticles at a concentration of 1 mg/mL for 120 h of multimodal imaging, including *ex vivo* MPI and NIR fluorescent imaging for 48 h.

### Western blot

After the diseased heart was cut into small pieces, a tissue lysate containing a protease inhibitor was added for lysis, and the supernatant was collected after centrifugation. The protein concentration was detected using a Pierce BCA Protein Assay Kit. A total of 20 µL of the G protein solution was mixed with sodium dodecyl sulfate (SDS) loading buffer solution, and a 4-15% polyacrylamide gel was used for electrophoresis separation. The reaction conditions as follows were maintained at 100 V for 90 to 120 min. When the bromophenol blue dye ran out of the polyacrylamide gel, electrophoresis was stopped and the protein was transferred to a polyvinylidene difluoride (PVDF) membrane. The PVDF membrane was treated in a sealed solution at room temperature for 1 h, and then overnight at 4 °C in a sealed solution containing a diluted primary antibody (1:1000 dilution ratio). The PVDF membrane was rinsed three times with TBS with Tween-20 (TBST) on a decolorization shaker at 25 °C for 15 min each time. The PVDF membrane was treated in a sealed solution containing a diluted secondary antibody (1:5000 dilution ratio) for 1-2 h at room temperature (20 °C) and then washed three times with TBST on a decolorization shaker at room temperature. Subsequently, chemiluminescence reaction detection was performed for 10 min per reaction, with the corresponding results being recorded and analyzed using Image J.

I/R H9c2 cells were lysed by adding tissue lysate containing a protease inhibitor. After centrifugation, the supernatant was obtained, and the protein concentration was detected by using the Pierce BCA Protein Assay Kit according to the methods described in the previous paragraph.

### Confocal microscopy imaging

The targeting ability of CRT peptides to TfR1 was detected by a confocal laser scanning microscope (CLSM). To achieve this goal, we treated the rat cardiac cell lines H9c2 and I/R H9c2 with TfR1 antibody to ensure that the antibody binds to the TfR1 site on the cell surface and blocks it. Then, we used Cy5 tag CRT (Cy5-CRT; Red) treating TfR1 antibody blocking and non-TfR1 antibody blocking H9c2 and I/R H9c2 for targeting experiments. The cells were also subjected to nuclear (DAPI; Blue) and cytoskeleton (FITC-phalloidin; green) staining to allow more accurate visualization of the targeting effect of CRT peptides.

Probe uptake was evaluated for H9c2 and I/R H9c2 cells using cell immunofluorescence of CLSM according to the procedures described above. We used Cy5 tag SCIO NPs (SCIO-Cy5; red), namely SCIO-Cy5, SCIO-Cy5-CRT, SCIO-Cy5-CPPS, and SCIO-Cy5-CRT-CPPS NPs for treating H9c2 and I/R H9c2 cells. In addition, the cells were subject to nuclear (DAPI; blue) and cellular lysosomes (lyso-tracker; green) standing to show the uptake of NPs by cellular lysosomes more accurately.

### Tetrazolium staining

Euthanasia was performed in the experimental mice, and the thorax was dissected to remove the heart and preserve the aorta. The blood in the heart was cleared in saline solution at 4 °C, dried the heart surface with gauze, and stored the heart in a refrigerator at -20 °C. After completion of cryofixation, hearts were placed in a microtome and five 1 mm thick sections were obtained from the apex. Approximately 5 mL of TTC phosphate buffer (pH 7.4) was prepared, and the sections were immersed in TTC phosphate buffer at 37 °C to allow adequate reaction for 15 min. Finally, photographs of both sides of each myocardial tissue were obtained using a high-resolution dissecting microscope. The infarction area appeared white, whereas the non-infarction area was bright red after TTC staining.

### Hematoxylin-eosin staining

The mouse heart tissue was cleaned three times with PBS, and fixed with a 4% paraformaldehyde solid solution. After the tissue was fully fixed, the diseased heart tissue was embedded using the paraffin embedding method, and the embedded diseased heart tissue was sliced using a paraffin tissue slicer with a thickness of 5 µm. For the hydration after deparaffinization, disc xylene I, xylene II, absolute ethanol I, absolute ethanol II, 95% alcohol for 5 min, 90% alcohol for 5 min, 80% alcohol for 5 min, and 70% alcohol embedding were used and washed with distilled water for 5 min. Harris hematoxylin staining was used for 3-8 min, rinsed with tap water, fractionated with 1% saline for a few seconds, then rinsed with tap water, rinsed with 0.6% ammonia water to restore the blue color, and rinsed with tap water again. Sections were then stained in an eosin staining solution for 1-3 min. The slices were placed in 95% alcohol I for 5 min, 95% alcohol II for 5 min, absolute ethanol I for 5 min, absolute ethanol II for 5 min, absolute ethanol II for 5 min, xylene I for 5 min, and xylene II for 5 min, and then dehydrate until transparent. The slices were removed from the xylene, slightly air-dried, and sealed with neutral gum. Microscopic examination, image acquisition, and analysis were performed thereafter.

### Prussian blue staining

Organs and tissues were fixed in 4% paraformaldehyde solid solution, and subjected to routine dehydration and embedding. Samples were sliced to thicknesses of 4 μm. Conventional dewaxing to water was performed. The mixture was then washed with distilled water for 1 min. The sections were sliced, stained with Prussian blue dye, and immersed for 15-30 min. The samples were washed thoroughly with distilled water for 5-10 min. The eosin staining solution was injected, and the nucleus was stained for 15-30 s. Samples were then rinsed with tap water for 1-5 s, and conventional dehydration-transparent neutral gum sealing followed. The slice scanner scanned and statistically analyzed the uptake of magnetic nanoparticles via histiocytes using Image J 1.53k.

### Immunohistochemistry

Pathological heart tissue sections were dewaxed and hydrated using xylene and various concentrations of ethanol. The tissue sections were then heated in sodium citrate buffer to restore antigen activity. After cooling to room temperature, the sections were treated with a 1% H_2_O_2_ solution for 20 min to block endogenous peroxidase activity. The sections were then washed twice with PBS and treated with Triton-X 100-Tris buffer before being treated with 10% bovine serum protein solution for 15 min at room temperature. Then, the sections were rewashed with PBS thrice, and then samples were sliced and put into a wet box at room temperature for 20 min. After drying without washing, an appropriately diluted primary antibody was added dropwise. Samples were refrigerated overnight at 4 °C. Samples were soaked in PBS thrice for 3 min, the excess liquid on the slice was dried, and then the diluted fluorescent secondary antibody was added dropwise. Samples were treated in a wet box at 37 °C for 1 h, and treated with PBS thrice. Starting from the addition of fluorescent secondary antibodies, all subsequent steps were performed in the dark when possible. Nuclei were re-stained, and the samples were treated in the dark after adding DAPI dropwise for 5 min. Nuclei were again re-stained, and excess DAPI was washed with PBS four times. An anti-fluorescent quenching sealing agent was used for sealing and fluorescent microscope observation.

### Cellular immunofluorescence

Each well was prefixed with 200 µL medium and 200 µL of 4% paraformaldehyde for 5 min. Thereafter, each well was fixed with 400 µL of 4% paraformaldehyde for 10 min. The waste solution was washed once with PBS, and 1 mL of PBS was added to each well. For breaking the film, the PBS was discarded, 1mL of PBS was added to the wash, and then it was also discarded. Next, 0.3% Tritonx-100 (diluted in PBS) was added to each well, and the plate was treated at room temperature for 5 min. Samples were washed with PBS for 3 × 5 min and PBS was discarded. For sealing, 300 µL of normal sheep serum (1 mL sheep serum + 9 mL PBS dilution) was added to each well and treated at room temperature for 1 h. After discarding the sealing solution, the samples were not immediately washed with PBS. Experimental antibodies were prepared according to the instructions, adding primary antibodies to the cells, and refrigerated overnight at 4 °C. Following washing with PBS thrice for 5 min, solutions were discarded. Darkroom conditions were used when adding the secondary antibody. Secondary antibodies were diluted at ratios of 1:200 and placed in a refrigerator at 4 °C. The secondary antibody was discarded, washed twice with PBS for 5 min, and discarded. For nuclear re-staining, DAPI was diluted to 1:1000 in a dark room, treated at room temperature for 7-10 min, washed with PBS thrice for 5 min, discarded, and using a fluorescent microscope to observe.

### MPI measurements

A Momentum MPI scanner with a magnetic field gradient strength of 6 T/m was used for analysis. Mice with intravenous injection of CON/CCI NPs were scanned using a Momentum MPI scanner at an excitation field (a peak amplitude along the z-axis: 20 mT at 45 kHz). Mice injected with 100 μL of 1mg/mL CON/CCI NPs were anesthetized using the mixed solution of 10 mg/kg xylazine and 100 mg/kg ketamine before scanning and imaging. Subsequently, the imaging of 2D MPI was scanned before probe injection and after 0, 3, 12, 24, 48, and 120 h. The field of view of the MPI scanner was 6 × 10 cm. The scan mode was isotropic and the scanning time was 3 min. For the imaging of 3D-MPI, the images were obtained with a field of view of 6 × 6 × 10 cm (scan mode: isotropic), taking 35 projections over a scanning time of 0.5 h. Quantifying the MPI signal was used to analyze the mice-injected CON/CCI NPs intravenously. The MPI signal was the average MPI signal within the region of interest of the heart (ROI). To obtain the MPI images of MI/R injury, the MI/R mice were sacrificed 48 h post-injection. Instantly scanning two-dimensional (2D) MPI images we used the following parameters: 6 x 10 cm field of view. Scanning pattern = isotropic. Parameters of the three-dimensional (3D) MPI image are field of view = 6 × 6 × 10 cm. Scanning pattern = isotropic. Projection repetitions = 35 times, total time 55 min. MPI images were analyzed using VivoQuant v.4.0 (Invicro, Boston, MA, USA).

### MPI/CT co-registration

CCI NPs (3 µL, 1 mg/mL Fe) in a plastic tube were placed as a reference marker and attached to the sample bed to align the MPI images with the 2D and 3D-computed tomography (CT) images. The 3D-MPI/CT images were strictly co-registered using VivoQuant.

### Statistical analysis

Data were expressed as mean ± standard deviation (SD). Comparisons were performed by paired t-tests within the group. Among groups, comparisons were performed by one-way analysis of variance (ANOVA) with multiple comparisons or *t*-tests when appropriate. A difference of *p* < 0.05 was considered significant.

## Results

### Synthesis and characterization of CCI NPs

**Figure 1:**
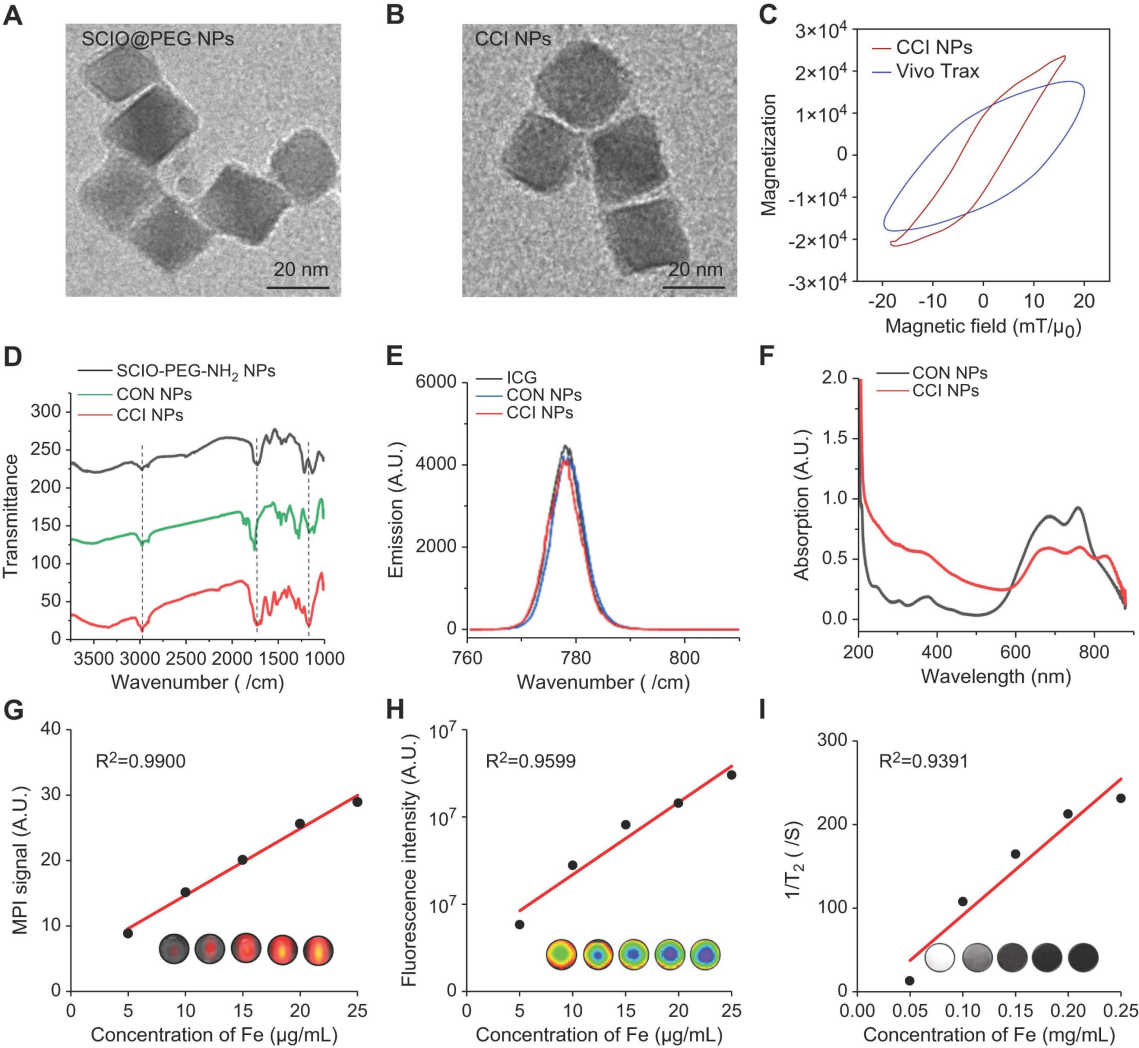
**Characterization of CCI NPs.** Transmission electron microscopy images of (A) SCIO@PEG NPs (Scale bar = 20 nm) and (B) CCI NPs (Scale bar = 20 nm). (C) The magnetization curves of CCI NPs and Vivo Trax. (D) FTIR spectra of SCIO@PEG-NH_2,_ CON NPs, and CCI NPs. (E) Fluorescent and (F) the UV-Vis absorbant spectra of CON NPs and CCI NPs. (G) MPI signals of CCI NPs increased with rising Fe concentrations. (H) The fluorescent intensity of CCI NPs increases with increasing fluorophore concentration. (I) T2 weighted MR images of CCI NPs at different iron concentrations.

To evaluate the *in vivo* biodistribution of the TfR1-targeting and CPPs-functionalizing NPs, CON, CRT, and CCI NPs were synthesized. Under transmission electron microscopy (TEM) images, CCI NPs are uniform cubes with good monodispersity and there was no significant difference in morphology between CON and CCI NPs (Figure 1A-B). Compared with Vivo Trax (the standards used in the MPI field) (Magnetic Insight Inc., USA), CCI NPs exhibited higher magnetization, lower relaxation effect, and smaller hysteresis loop in the middle opening of the curve, demonstrating excellent performance of the synthesized probes (Figure 1C). The hydrated particle sizes of CON, CRT, and CCI NPs are shown in Figure S1A, and the zeta potential values are -5.71 ± 0.67, -6.23 ± 0.16, and -7.61 ± 1.02 mV, respectively (Figure S1B). The alterations in dynamic light scattering (DLS) analysis and zeta potential among CON, CRT, and CCI NPs reflected that of the combination of CRT and CPPs, where the particle size of the probe gradually increased, but it was still within the acceptable range. At the same time, the change of zeta potential also indicates the change of charge properties on the surface of the probe after its binding to CRT and CPPs. There was also no statistically significant difference in the polydispersity (PDI) measures of the probe after 1 week of measurement (Figure S1C). In Fourier transform infrared (FTIR) spectra (Figure 1D, S1D), CCI NPs showed a characteristic ether and indicative amide bond peaks and were compared with PEG2000, CPPs, ICG-NHS, and CON NPs, confirming the successful binding of ICG and targeted peptide-SMCC to SCIO-NPs. The fluorescent emission spectra of CON and CCI NPs were similar with an emission maximum at 820 nm, indicating a near-infrared capability (Figure 1E). Furthermore, the fluorescent properties of ICG were not altered by binding to SCIO NPs. After labeling with CRT and CPPs, the UV-Vis absorption spectrum of CCI NPs changed and was enhanced at approximately 230 nm (Figure 1F). The MPI and NIR properties of different concentrations of CCI NPs are important for detecting TfR1 *in vivo*. The MPI signals (y = 1.017x + 4.512, R^2^ = 0.9900, *p* < 0.001) of the CCI NPs and the NIR intensity (y = 0.166×10^6^x + 1.017×10^6^, R^2^ = 0.9599, *p* < 0.01) showed a linear dependence with the iron concentration (Figure 1G-H). Furthermore, the Transverse (T2)-dependent MR of nanoparticles depends on the sample concentration and decreases with increasing contrast agent concentration (Figure 1I). These properties suggest that the probe CCI NPs synthesized here can transduce NIR, MPI, and MRI signals in a concentration-dependent manner. This makes them suitable for observing MI/R *in vivo*. The observations demonstrated that the synthesized probe CCI NPs exhibited strong MPI/MRI/NIR imaging performance, making it suitable for multimodal imaging.

### *In vitro* targeting specificity and cytotoxicity of CCI NPs

**Figure 2:**
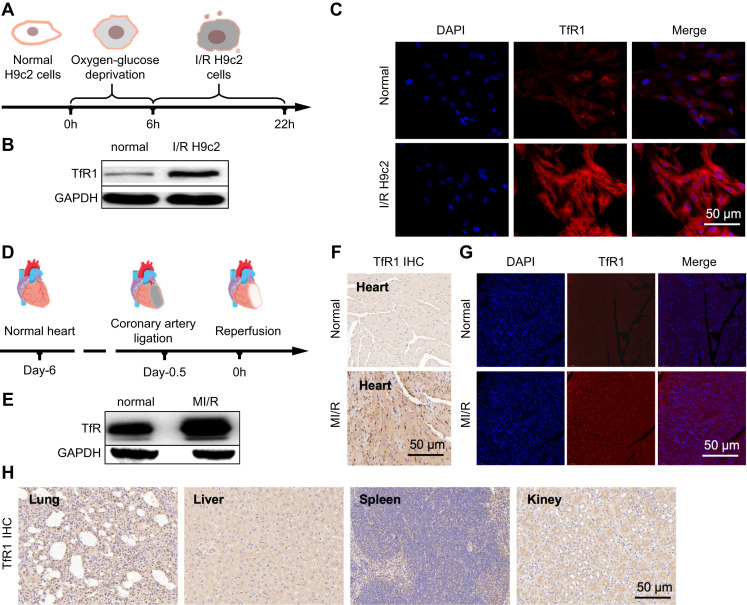
**Examination of TfR1 expression and targeting specificity in I/R H9c2 cells or MI/R mouse.** (A) Schematic diagram of the I/R H9c2 cell model construction process. (B) Expression of TfR1 in normal and I/R H9c2 cells was detected via western blot. (C) Immunofluorescence staining shows high expression of TfR1 in the I/R H9c2 cell model, but not in normal H9c2 cell lines (Scale bar = 50 µm). (D) Schematic illustration of the MI/R mouse model building process. (E) Expression of TfR1 in normal and MI/R mouse models. (F) A high level of TfR1 expression was detected in the heart tissue of the MI/R mouse model by immunohistochemical staining (Scale bar = 50 μm). (G) A high level of TfR1 expression was detected in injured heart tissue of the MI/R mouse model by immunofluorescent staining (Scale bar = 50 μm). (H) Immunohistochemical staining showing the expression of TfR1 in other normal organs of the MI/R mouse model (Scale bar = 50 μm).

Previous studies showed a low expression of GPX-4 and SLC7A11 in ferroptosis. Compared to untreated cells in the control group, western blot (Figure S2A-C) and immunofluorescence (Figure S3A-C) of the control (H9c2) and model (I/R H9c2) showed a down-regulated trend in their expression. Moreover, compared with the mice in the normal group, the expression of GPX-4 and SLC7A11 decreased in the western blot of mice in the MI/R model group (Figure S4A-C). Furthermore, to validate the iron binding of TfR1 as a specific target for MI/R imaging, this property was analyzed in I/R H9c2 cell lines and heart tissues. In H9c2 cell lines with oxygen-glucose deprivation and reoxygenation (Figure 2A), high TfR1 expression levels were detected, and their expression was further analyzed using western blot and immunofluorescence. Both techniques indicated that TfR1 was overexpressed in I/R H9c2 cells (Figure 2B-C, S5A-B). Thereafter, the MI/R mouse model was constructed by ligating the anterior descending branch of the coronary artery for 30 min before the ligature was removed to restore cardiac perfusion, and the anterior descending branch of the coronary artery was ligated (Figure 2D). Similarly, the expression of TfR1 was observed by western blot, immunofluorescence, and immunohistochemistry, revealing its high expression in heart tissues of the MI/R mouse model (Figure 2E-G, S5C-E). TfR1 was also found to be highly expressed in MI/R heart tissue but not in the lungs, liver, spleen, and kidneys (Figure 2H).

**Figure 3:**
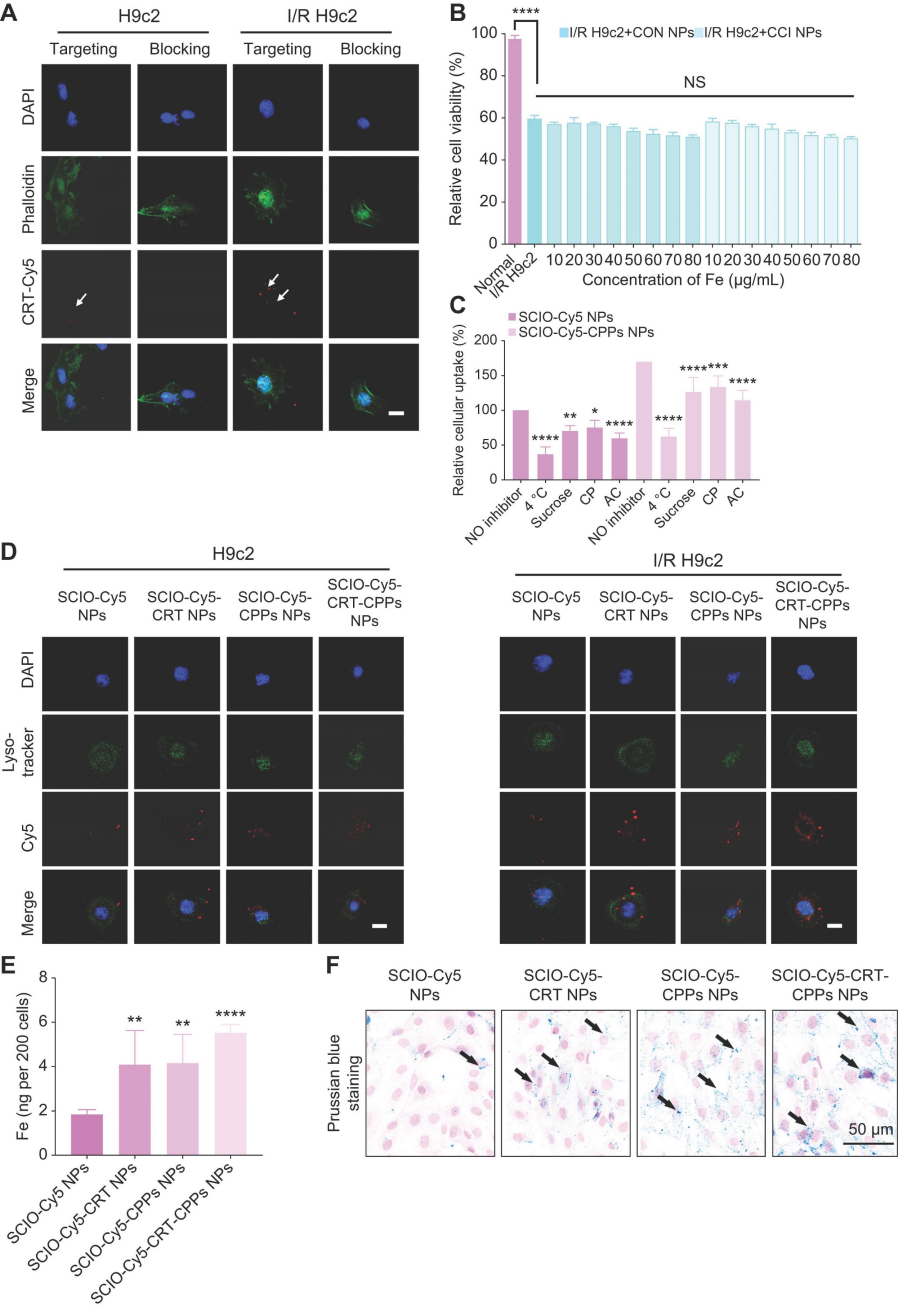
** Validation of the specificity and uptake of CRT/CPPs functionalized NPs on H9c2 cells.** (A) CLSM images of the binding affinity of Cy5-CRT (red). FITC-phalloidin (cytoskeleton marker, green) and DAPI (nucleus marker, blue) were used to stain H9c2 or I/R H9c2 cells, with or without TfR1 antibody pre-incubation. Scale bar = 20 μm. (B) Relative cell viability of H9c2 and I/R H9c2 cells treated with various concentrations of CON/CCI NPs for 24 h (n = 6; one-way ANOVA; NS, not significant; *p < 0.05, **p < 0.01, ***p < 0.001, and ****p < 0.0001). (C) The effects of different inhibitors on the internalization of CON/CCI NPs were measured using flow cytometry after incubation of H9c2 cells at 37 °C for 4 h. Incubation at 4 °C inhibits endocytosis. sucrose, chlorpromazine (CP), and ammonium chloride (AC). Normalized fluorescent intensity of cells treated with SCIO-Cy5 NPs without inhibitor at 37 °C (n = 6; one-way ANOVA; NS, not significant; *p < 0.05, **p < 0.01, ***p < 0.001, and ****p < 0.0001). (D) Representative confocal images of H9c2/IR H9c2 cells treated with SCIO-Cy5/SCIO-Cy5-CRT/SCIO-Cy5-CPPs/SCIO-Cy5-CRT-CPPs NPs (red) for 6 h. Lyso-tracker (lysosomes marker; green) and DAPI (nucleus marker; blue) were used to stain H9c2 or I/R H9c2 cells. The third row shows magnified details to better demonstrate the presence of NPs. Scale bar = 20 µm. (E) The content of cellular iron in normal H9c2 cells was analyzed using ICP-MS after the treatment of SCIO-Cy5/SCIO-Cy5-CRT/SCIO-Cy5-CPPs/SCIO-Cy5-CRT-CPPs NPs, respectively. (n = 6; one-way ANOVA; NS, not significant; *p < 0.05, **p < 0.01, ***p < 0.001, and ****p < 0.0001). (F) Prussian blue staining of H9c2 cells after co-incubation with SCIO-Cy5/SCIO-Cy5-CRT/SCIO-Cy5-CPPs/SCIO-Cy5-CRT-CPPs NPs (Scale bar = 50 μm).

The enlarged portion of the third row of Figure 3A shows the presence of CRT-Cy5 (white arrows). In addition, the targeting ability of the probes was tested as well. After co-incubation with TfR1 antibody, the uptake of CRT-Cy5 by I/R H9c2 cells decreased, compared with I/R H9c2 cells without TfR1 antibody co-incubation (Figure S6A). In the targeting group, the uptake of Cy5-CRT by I/R H9c2 cells was significantly higher than that of normal H9c2 cells, confirming the CRT peptides-specific cell-targeting ability (Figure S6A). The evaluation of I/R H9c2 cytotoxicity before *in vitro* administration showed a 40% reduction in cell count after I/R treatment (Figure 3B). Furthermore, CCK assays measuring the cytotoxic effect of I/R H9c2 cells revealed no marked cytotoxicity following incubation with CON/CCI NPs across various concentrations within 24 h, with a relative cell viability > 50% at concentrations ≤ 80 mg/mL; thus, no significant cytotoxicity was observed, thereby indicating low toxicity.

To investigate the interaction of nanoparticles with H9c2 cells, the mechanism of cellular internalization was investigated. Instead of ICG, the nanocarriers were loaded with Cy5 fluorescent dye with or without CPPs (as described above). The performance of the obtained nanoparticles (SCIO-Cy5-CPPs-NPs and SCIO-Cy5-NPs) was comparable to that of ICG-loaded nanoparticles. Compared to 37 °C, the incorporation of both nanoparticles by H9c2 cells was significantly inhibited (>63%) at 4 °C (Figure 3C), suggesting that the internalization of these nanoparticles was largely via energy-dependent rather than energy-independent endocytosis. Using specific inhibitors of various endocytic signaling pathways on H9c2 cells (Figure 3C), the uptake of nanoparticles by H9c2 cells was reduced by over 25% with the use of sucrose and the inhibitor of clathrin-coated vesicles. In addition, the involvement of endosomes/lysosomes in cellular uptake was also demonstrated with the agent of lysosomes. We observed a more than 40% reduction in the uptake of SCIO-Cy5 NPs and SCIO-Cy5-CPPs NPs (Figure 3C), via using AC, suggesting that these compartments were very important for nanoparticle entrapment. Furthermore, endosomal transport may involve the transport of intact particles across cellular barriers, while lysosomes may be involved in particle degradation and drug release. Fluorescence staining was performed on I/R and normal H9c2 to examine lysosomal colocalization with nanoparticles, finding that after 6 h of co-incubation, when normal and I/R H9c2 cells were treated with SCIO-Cy5-CRT/SCIO-Cy5-CPPs/SCIO-Cy5-CRT-CPPs, the Cy5 signal was more localized near or within lysosomes compared with SCIO-Cy5, confirming the ability of targeted-nanoparticles to reach these cells, and suggesting the presence of lysosomal absorption (Figure 3D, S6B). Comparatively, the enrichment of target nanoparticles modified with CPPs-CRT was stronger.

Cellular internalization was then studied using inductively coupled plasma-mass spectrometry (ICP-MS) and Prussian blue staining. SCIO-Cy5-CRT-CPPs, SCIO-Cy5-CPPs, SCIO-Cy5-CRT, and SCIO-Cy5 NPs cell internalized 5.54 ± 0.36, internalized 4.17 ± 1.28, 4.11 ± 1.54, and 1.85 ± 0.21 ng Fe, suggesting the enrichment of nanoparticles targeting CPPs modification was stronger (Figure 3E). In Prussian blue staining of H9c2 cells, SCIO-Cy5 NPs showed almost no cell staining, whereas SCIO-Cy5-CRT-CPPs NPs displayed a large number of stained myocardial cells. Thus, the uptake of nanoparticles by myocardial cells was enhanced through CPPs and CRT modification (Figure 3F). Additionally, Prussian blue staining semiquantitative analysis showed that the nanoparticle retention of modified CPPs or CRT was significantly increased compared with unmodified CPPs or CRT, indicating that CPPs and CRT enhanced the efficiency of cardiomyocyte uptake of nanoparticles (Figure S7). Accordingly, the probe displayed strong specificity, low toxicity, and high uptake efficiency, making it suitable for targeting TfR1 in feMPI and multimodal imaging. It can thus be widely used for noninvasive detection of ferroptosis-related diseases.

### FeMPI in MI/R mouse model

**Figure 4:**
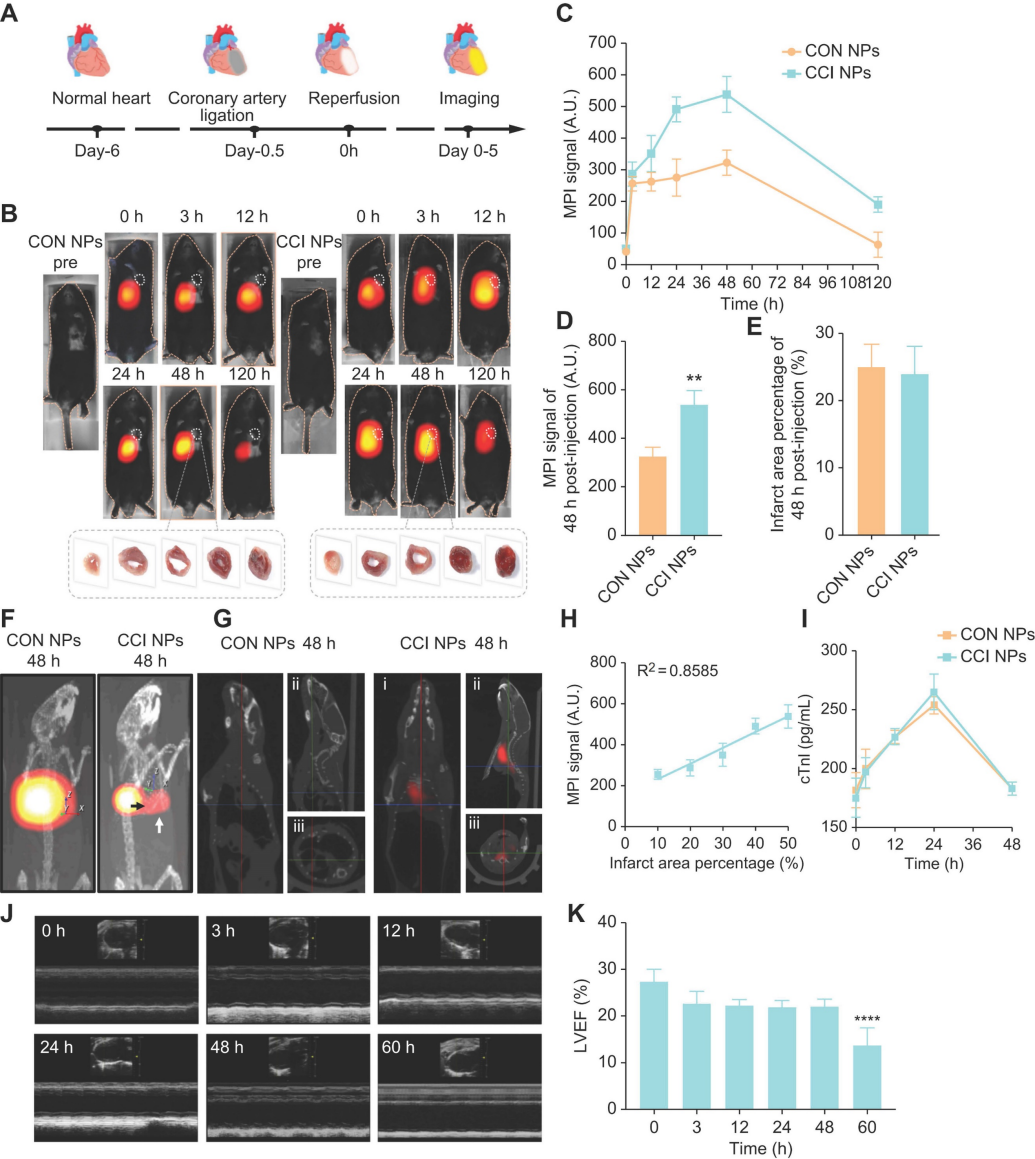
***In vivo* feMPI imaging and echocardiography of a mouse MI/R model.** (A) Schematic of the construction and multimodal imaging of MI/R mouse model. (B) *In vivo* MI/R mouse model from different groups (CON NPs *vs.* CCI NPs) at different time points (0, 3, 12, 24, 48, and 120 h) of MPI image. White dotted circles indicate the location of the heart (n = 3). TTC staining images of heart tissue from each group at 48 h post-injection are shown at the bottom of the image. (C) Quantitative comparison of MPI signals of CON/CCI NPs (n = 3). (D) The 48 h post-injection cardiac MPI signal (white dotted circle) was compared for different groups (CON NPs *vs.* CCI NPs; n = 3; Student's t-test. *p < 0.05, **p < 0.01, ***p < 0.001, and ****p < 0.0001). (E) Comparison of the percentage of infarct area stained by TTC in each group at 48 h (CON NPs *vs.* CCI NPs; n = 3; Student's t-test. *p < 0.05, **p < 0.01, ***p < 0.001, and ****p < 0.0001). (F) 3D-MPI/CT image. The black arrow points to the clear boundary between the MPI signal in the heart and the liver, whereas the white signal points to the MPI signal in the heart. (G) 3D-MPI/CT images (i: coronal plane; ii: sagittal plane; iii: horizontal plane) of the CON/CCI NPs without the liver at 48 h. (H) The linear relationship between MPI signal and percent infarct area stained by TTC. (I) Changes in cTnI within 48 h (CON NPs *vs.* CCI NPs; n = 3). (J) Echocardiograms at different time points in the CCI NPs (0, 3, 12, 24, 48, and 60 h). (K) Analysis of LVEF at different times (0, 3, 12, 24, 48, and 60 h) in the CCI NPs (n = 3; one-way ANOVA, *p < 0.05, **p < 0.01, ***p < 0.001, and ****p < 0.0001).

The anterior descending coronary artery ligation of mice, removed the ligature to restore cardiac perfusion after 30 minutes and probes were injected via the tail vein for MPI/NIR/MRI (Figure 4A). FeMPI was performed in an *in vivo* MI/R mouse model. After tail vein injection of CON/CCI NPs (n = 3 for each), changes in the MPI signal were observed after injection at 0, 3, 12, 24, 48, and 120 h (Figure 4B). Various groups of cardiac tissue images stained with TTC at 48 h post-injection were displayed. Compared to CON NPs, the MPI signal of CCI NPs peaked at 48 h and persisted for 120 h (Figure 4C). In Figure 4D, the analysis showed that despite the interference of the liver background, the CCI NPs group displayed a significant difference in MPI signals in the heart compared with that of the CON NPs group. However, it showed no significant difference in the infarct area percentage stained by TTC (Figure 4E) and Masson staining (Figure S8A-B).

Furthermore, 3D-MPI/CT revealed a denser distribution of CCI NPs (Video S1) than that of CON NPs in the heart (Video S2; Figure 4F), with spatial position details observed across the entire body. In addition, as shown in Figure 4G, compared with CON NPs, there was a significant MPI signal area of the heart without the liver in the CCI NPs group at 48 h in the coronal plane, sagittal plane, and horizontal plane (Video S3). The infarct area percentage stained by TTC revealed a linear relationship between MPI signals (*y* = 7.707*x* + 153.5, R^2^ = 0.8585, *p* < 0.0001) and the cardiac injury area; thus, it was concluded that feMPI can quantitatively detect the degree of MI/R cardiac injury (Figure 4H). These feMPI results were consistent with those of the commonly used clinical indicator cTnI in MI/R within the first 24 h (Figure 4I). Ultrasound measurements showed a significant difference in ventricular ejection fraction (LVEF) between 0 and 60 h after IR (Figure 4J). *In vivo*, feMPI has shown great feasibility in accurately detecting MI/R-induced cardiac injury approximately 48 h earlier than the echocardiography (Figure 4K). Collectively, CCI NPs could serve as an efficient feMPI contrast agent for the quantitative, early, and noninvasive evaluation of ferroptosis-related cardiac reperfusion injury in the MI/R mouse model.

### NIR/MRI multi-modality imaging in MI/R mouse model

**Figure 5:**
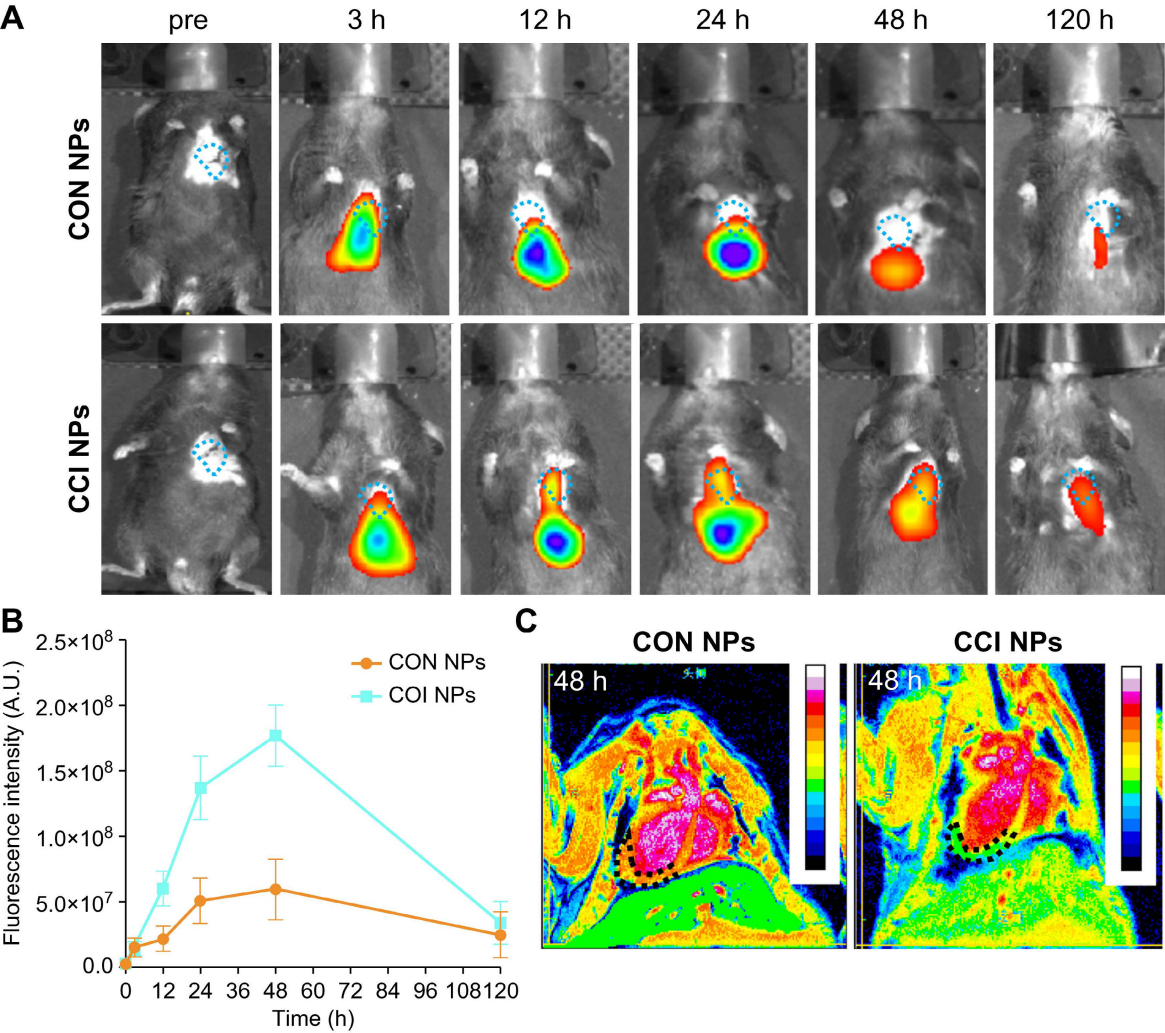
***In vivo* NIR/MRI multimodal imaging of the MI/R mouse model.** (A) NIR fluorescent images of the MI/R mouse model at different time points *in vivo* from different groups (CON NPs *vs.* CCI NPs; n = 3). (B) Quantitative comparison of fluorescence intensities of CON/CCI NPs (n = 3). (C) Images of the MI/R mouse model injected CON/CCI NPs obtained from MRI at 48 h post-injection (n = 3). The location of the cardiac tissue with a locally observable low intensity is circled by black dots.

When using NIR/MRI to simultaneously monitor the MI/R model, NIR data showed that CON and CCI NPs produced the highest fluorescence intensity in the heart 48 h after tail vein injection (Figure 5A). Compared with CON NPs, the fluorescent intensities of CCI NPs were significantly enhanced, and those of CCI NPs were 2.96-fold that of CON NPs at 48 h post-injection (Figure 5B). The fluorescent intensity of CCI NPs persisted for 120 h. The NIR fluorescent data suggests that CCI NPs exhibit superior targeting capabilities towards the heart compared to CON NPs. Quantitative analysis of the fluorescent intensity data revealed stronger signals (Figure 5B). To further validate the imaging efficacy of CRT and CPPs NPs probe sets in the MI/R mouse model, we added NIR fluorescent imaging of the MI/R mouse model 48 h post-injection of CRT/CPPs NPs (Figure S9A), and the imaging results were statistically analyzed (Figure S9B). The NIR fluorescent intensity of the liver in CPPs and CCI NPs was enhanced compared with the CON group, proving that CPPs increased the background uptake. Compared with CRT NPs, the fluorescent intensity in the liver increased in the CCI NPs; the relative fluorescence intensity of heart in CCI NPs was 2.4-fold that of CPPs NPs and 2.36-fold of CRT NPs, indicating that although CPPs can increase the background uptake, the synergistic use of CPPs and CRT preferentially promotes the targeted binding of the NPs to the injured heart tissue under the background of high expression of TfR1 in MI/R. As a representative clinical imaging method, an MRI examination (Figure 5C) was also performed. The results indicated that the T2 weighted signals of the CCI NPs in the coronal position gradually spread to the entire MI/R cardiac tissue (Figure 5C); however, the MRI signals of the CON NPs group were limited to the liver. Simultaneously, MRI provided additional anatomical details to locate the position of the probes within the heart. We labeled and counted the MRI images. NPs enrichment was seen in cardiac lesions in CCI NPs and that was statistically different compared with CON NPs, as shown in Figure S9C.

**Figure 6:**
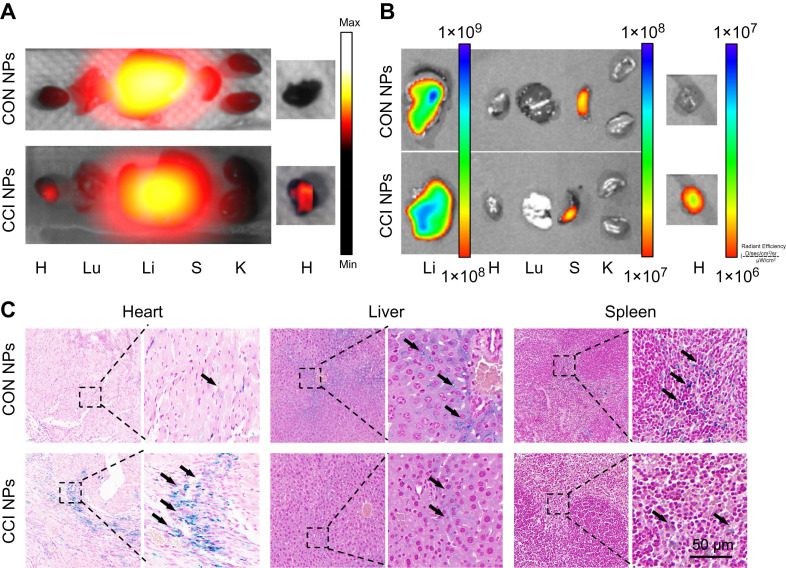
***Ex vivo* imaging and Prussian blue staining of the MI/R mouse model.** The *ex vivo* imaging for the heart and the other major organs of the MI/R mouse model, which were removed and captured 48 h after injection, were obtained using MPI (A) and NIR fluorescent imaging (B). H: Heart; Lu: Lung; Li: Liver; S: Spleen; K: Kidney. (C) Prussian blue staining of the resected MI/R hearts, livers, and spleens (Scale bar = 50 µm; the black arrows indicate positive iron staining).

To further support the observations made through* in vivo* optical magnetic multimodality imaging, we obtained the heart and major organs for *ex vivo* multimodal imaging. *In vitro* MPI/NIR images were acquired 48 h post-injection (Figure 6A-B). The fluorescent intensity of the heart was higher in CCI NPs than in the CON NPs. As shown in Figure. 6C, S10, there was more iron ion accumulation of the cardiac tissue in the CCI NPs than in the CON NPs group. This may be because the coupling of CRT to CPPs allowed the magnetic particles to penetrate the cell membrane and enter the cell interior, thereby increasing iron uptake. In contrast, the CON NPs group did not have efficient access to the cell interior and therefore had a relatively low iron ion intake. In addition, there were several sporadic areas of iron-positive staining in the liver and spleen tissues of the CON NPs and CCI NPs group. The biosafety evaluation of major organs by hematoxylin-eosin (H&E) staining of the mouse in the CCI NPs group and CON NPs group was performed. No abnormalities were detected in major organs such as the heart, liver, spleen, lungs, and kidneys between the CON NPs and CCI NPs group as illustrated in Figure S11A. Furthermore, the liver/kidney function of the mouse in the CCI NPs group, CON NPs group, and healthy mouse group was assessed. The liver function was evaluated through the measurement of alanine aminotransferase (ALT), aspartate aminotransferase (AST), and alkaline phosphatase (ALP) levels. Moreover, renal function was assessed by serum creatinine (CREA) and blood urea nitrogen (UREA), revealing no difference between either of the two groups and the healthy controls (Figure S11B). We also performed feMPI in a doxorubicin-induced mouse acute cardiac injuries model, and the results showed that feMPI could detect cardiac injuries by CCI NPs (Figure S12). In summary, NIR/MRI multimodal imaging can assist CCI NPs in providing more accurate detection of ferroptosis-mediated cardiac injury in an MI/R mouse model.

## Discussion

Eleven years after its discovery, ferroptosis has received increasing attention regarding the pathogenesis and progression of various common ailments, including cardiovascular diseases, tumors, and neurodegenerative diseases 31. Previous studies have shown that ferroptosis is associated with the early pathogenesis of heart injury and various ischemic injury-related diseases 9. Owing to the unique roles of iron overload and lipid peroxidation in MI/R injury, ferroptosis may be a therapeutic mechanism and potential cause for such ischemic diseases. This hypothesis has been validated in clinical trials, where some iron-chelating agents and antioxidants, such as deferoxamine and vitamin E, have shown potential clinical benefits for the treatment of MI/R diseases 32. The abnormal expression of TfR1 and the curative effect of iron homeostasis therapy in MI/R have been demonstrated in multiple animal models, linking MI/R-induced cardiac injury to ferroptosis caused by abnormal iron metabolism 33. The overexpression of TfR1 and changes in the transport of iron are crucial in ferroptosis-related heart lesions. Therefore, the present study hypothesized that molecular imaging targeting TfR1 may contribute to the accurate detection of MI/R injury; however, few molecular imaging methods have been reported for the noninvasive detection of ferroptosis in MI/R *in vivo*, hampering the development of drugs for ferroptosis treatment. Accordingly, this research further proposed the relevance of detailed iron homeostasis in healthy bodies. In addition, the overexpression of TfR1 can serve as a biomarker for ferroptosis.

MPI is an ideal noninvasive positive technique for *in vivo* imaging, as it is characterized by high sensitivity, and no penetration depth limitation nor any ionizing radiation 34. In addition, the enhanced specificity of MPI has shown great potential for the quantitative diagnosis of specific molecular biological targets *in vivo*. The present study designed a CCI NPs probe for feMPI based on targeting peptides, SCIO NPs, and ICG. The targeting peptides assist in enhancing the specific labeling and uptake of probes; therefore, the CCI NP probes provide two important advantages for feMPI: (i) enhanced MPI signal in target tissues; (ii) the signal intensity has a linear correlation with the percent infarct area stained by TTC staining, allowing for a quantitative assessment of the extent of heart damage. Previous studies have reported methods for visualizing ferroptotic molecules using fluorescence, such as PET or MRI 35,36. To the best of our knowledge, the novel research here has elaborated upon the specificity of green, safe materials and simple synthesis methods for preparing *in vivo* probes to visualize MI/R-induced cardiac injury using feMPI. Additionally, this probe can be used for MRI/NIR fluorescent imaging, thereby integrating the advantages of multimodal imaging techniques. It is anticipated that this non-invasive, accurate, and long-term imaging method will have great potential for clinical conversion.

FeMPI can quantitatively detect abnormal intracellular levels of TfR1 in MI/R-induced myocardial cell damage. Notably, the non-invasive dynamic monitoring results of feMPI are consistent with the measurements of cTnI, a commonly used clinical biochemical indicator of detection index for MI/R injury. These results suggest that changes in TfR1 occur more consistently than changes in serum biomarkers excreted from primary tissue, which may aid in the diagnosis of tissue-specific heart disease before imaging indicators 29,31. In addition, feMPI provides a powerful means of avoiding cardiac remodeling interference and quantitatively evaluating the degree of cardiac lesions following reperfusion. This method shows immense promise for accurately evaluating cardiac function after MI/R in real-time to efficiently adjust treatment deployment and improve treatment outcomes. Despite of the interference of the liver background, the CCI NPs group detected clear MPI signals in the heart through 3D-MPI/CT. Due to the insufficient resolution of MPI and the presence of interference of the liver background, it is difficult to visualize heart injury quickly and accurately through 2D-MPI. Therefore, we will further optimize the structure of the probes to reduce the non-specific uptake of probes by the liver, thereby improving the detection accuracy of MIR-induced heart damage by feMPI. Furthermore, loading drugs for the diagnosis and treatment of MPI-guided MI/R will be the direction of our future work. In addition, based on the outstanding performance of 3D-feMPI in displaying MI/R heart damage, it is reasonable to believe that this technique may also apply to other iron-related diseases, such as doxorubicin-induced cardiac injury, neurodegenerative diseases, and cerebral ischemia-reperfusion injury. This opens up new possibilities for the diagnosis and treatment of future diseases.

In summary, the present study reported a TfR1 and CPPs dual-targeted probe for the noninvasive quantitative evaluation of the degree of MI/R-induced cardiac injury *in vivo* using the feMPI strategy. It has shown great feasibility in accurately detecting MI/R-induced cardiac injury in vivo, with dual-targeted probes assessing MI/R-induced cardiac injury ≥ 48 h earlier than echocardiography, thereby compensating for the difficulty in detecting MI/R damage during cardiac remodeling. Moreover, the feMPI results here were consistent with cTnI, a commonly used clinical indicator of MI/R within the first 24 h. In addition, CCI NPs probes can enhance the MPI signal of MI/R-induced cardiac injury and specificity of MPI, extending the residence time of the lesion by 5 days compared to non-targeted probes, and permitting a longer observation time window for continuous evaluation of MI/R-induced cardiac injury. CCI NPs probes can be powerful tools for studying the process of ferroptosis *in vivo* through the feMPI strategy, providing clues for molecular imaging and drug development directed at the treatment of various ferroptosis-related diseases.

## Supplementary materials

Experimental procedures, synthetic details and characterization, additional analytical data; Figures S1-S12. 3D MPI/CT images of the MI/R mouse model incubated with CON and CCI NPs; Video S1-S2. Video of MI/R mouse model without the liver incubated with CCI NPs; Video S3. Supplementary figures and tables.
