# Supplementary material for: Quantitative visualization of myocardial ischemia-reperfusion-induced cardiac lesions via ferroptosis magnetic particle imaging
Source: Theranostics. 2024 Jan 1;14(3):1081–97. doi: 10.7150/thno.89190 (PMC10797296; doi:10.7150/thno.89190)
Supplement: Supplementary file 1 — Experimental procedures, synthetic details and characterization, additional analytical data; Figures S1-S12. 3D MPI/CT images of the MI/R mouse model incubated with CON and CCI NPs; Video S1-S2. Video of MI/R mouse model without the liver incubated with CCI NPs; Video S3. Supplementary figures and tables. [file thnov14p1081s1.zip › Supplementary figures.pdf]

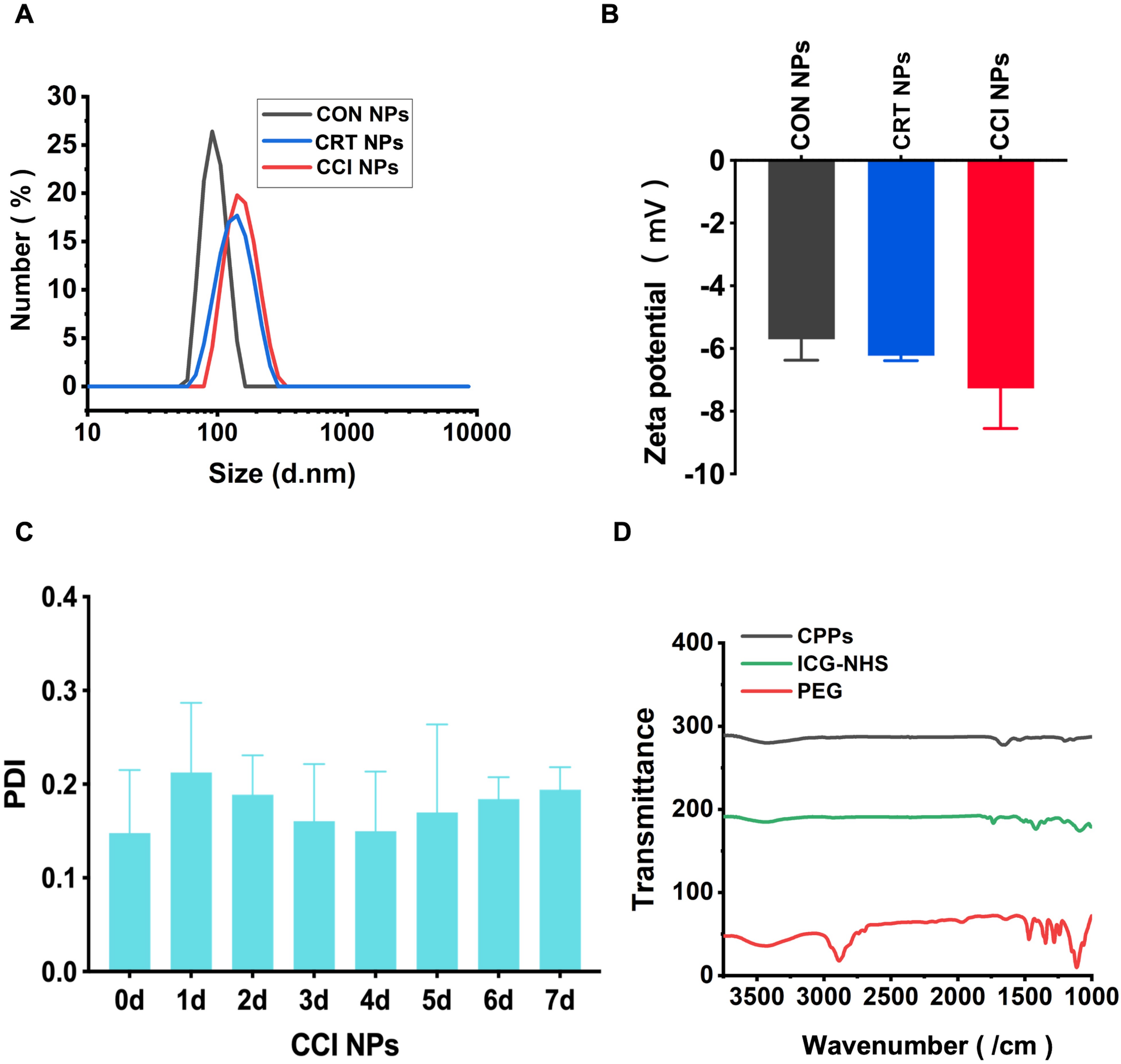

A
Number (%)
Size (d.nm)
CON NPs
CRT NPs
CCI NPs
30
25
20
15
10
5
0
10
100
1000
10000
B
Zeta potential (mV)
CON NPs
CRT NPs
CCI NPs
0
-2
-4
-6
-8
-10
C
PDI
0.4
0.3
0.2
0.1
0.0
0d
1d
2d
3d
4d
5d
6d
7d
CCI NPs
D
Transmittance
CPPs
ICG-NHS
PEG
400
300
200
100
0
3500
3000
2500
2000
1500
1000
Wavenumber (/cm)

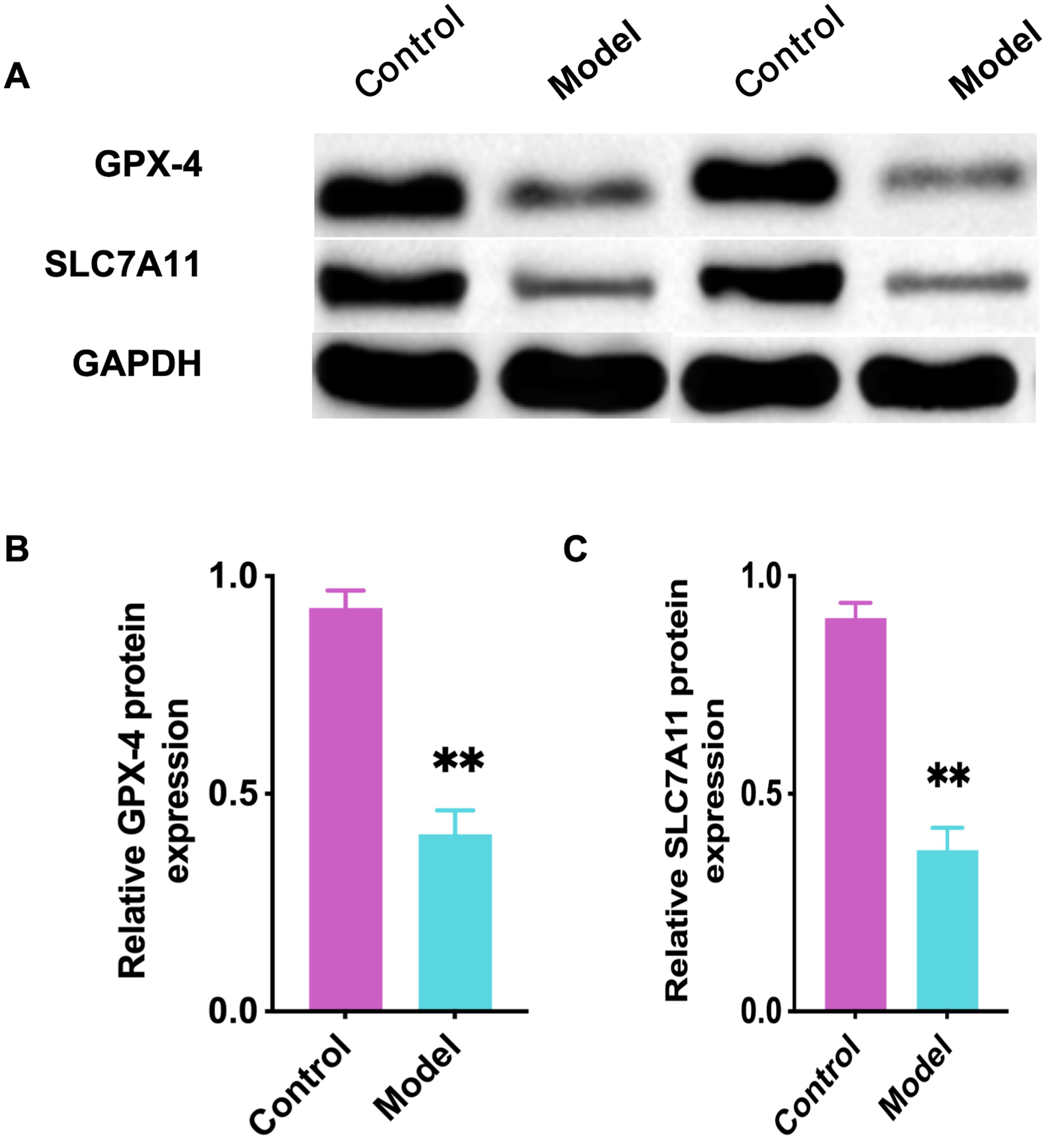
A
Control
Model
Control
Model
GPX-4
SLC7A11
GAPDH
B
Relative GPX-4 protein expression
1.0
0.5
0.0
**
Control
Model
C
Relative SLC7A11 protein expression
1.0
0.5
0.0
**
Control
Model

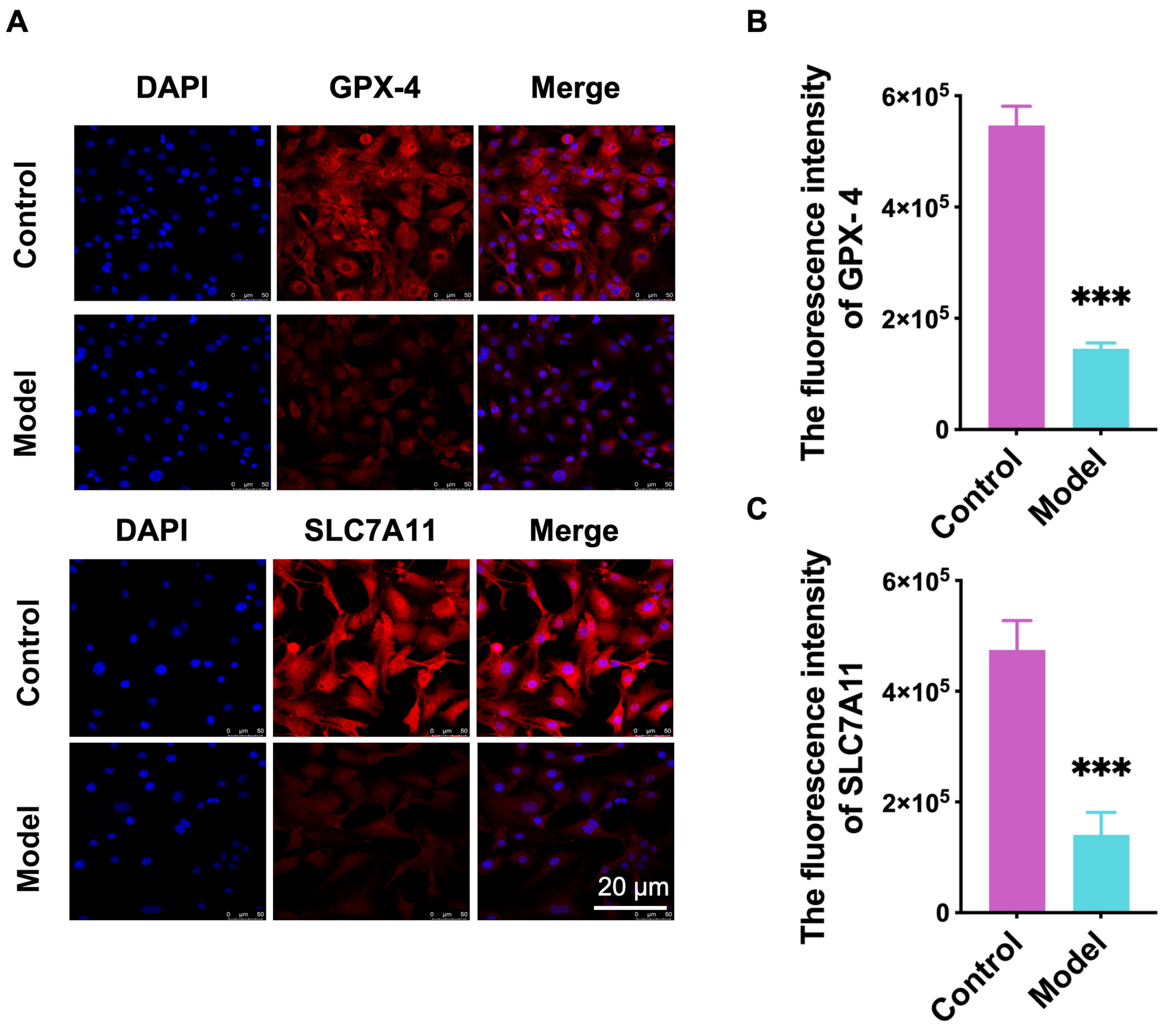
A
DAPI
GPX-4
Merge
Control
Model
DAPI
SLC7A11
Merge
Control
Model
20 μm
B
The fluorescence intensity of GPX- 4
6×10^5
4×10^5
2×10^5
0
***
Control
Model
C
The fluorescence intensity of SLC7A11
6×10^5
4×10^5
2×10^5
0
***
Control
Model

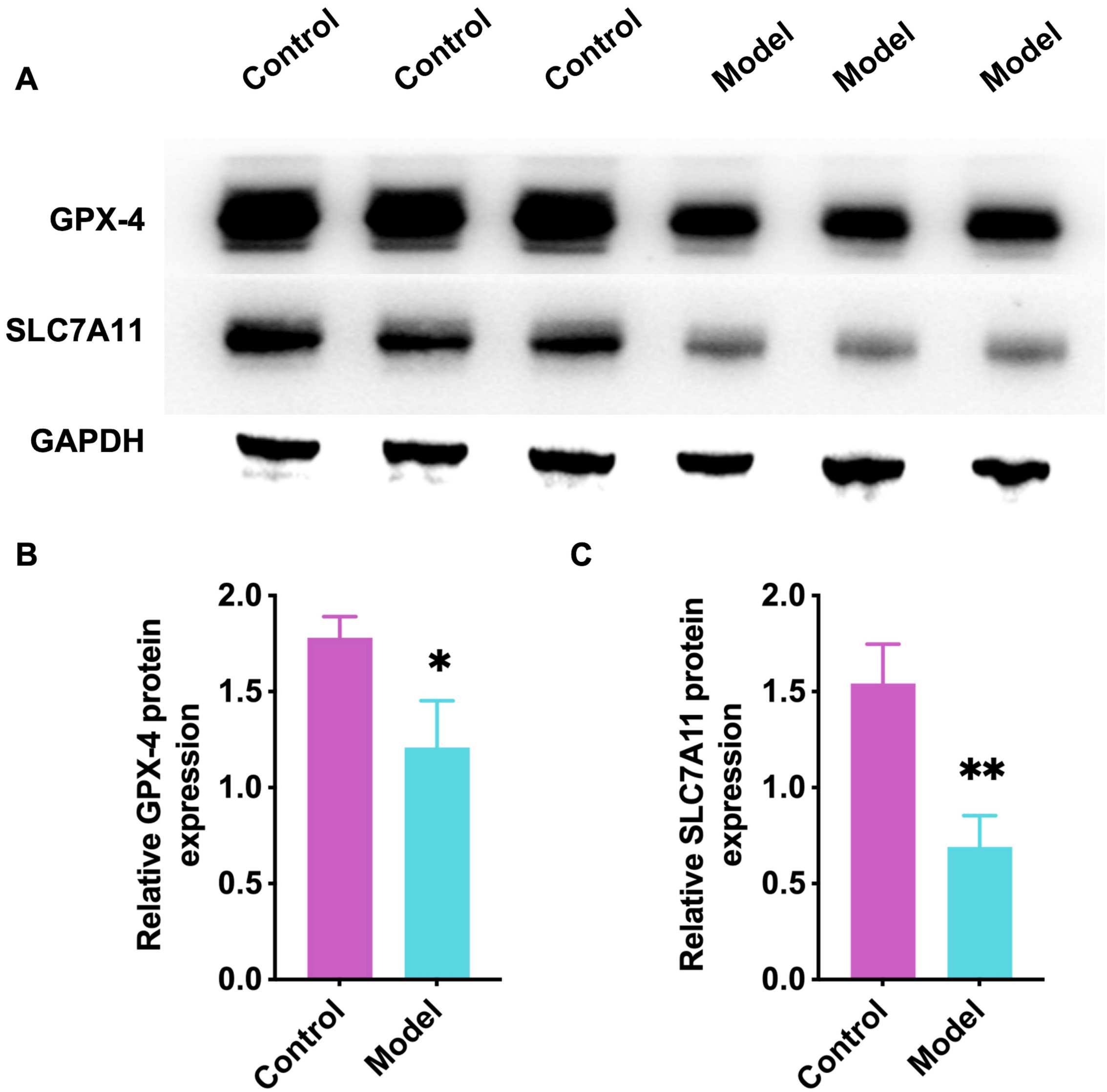
A
Control
Control
Control
Model
Model
Model
GPX-4
SLC7A11
GAPDH
B
Relative GPX-4 protein expression
2.0
1.5
1.0
0.5
0.0
*
Control
Model
C
Relative SLC7A11 protein expression
2.0
1.5
1.0
0.5
0.0
**
Control
Model

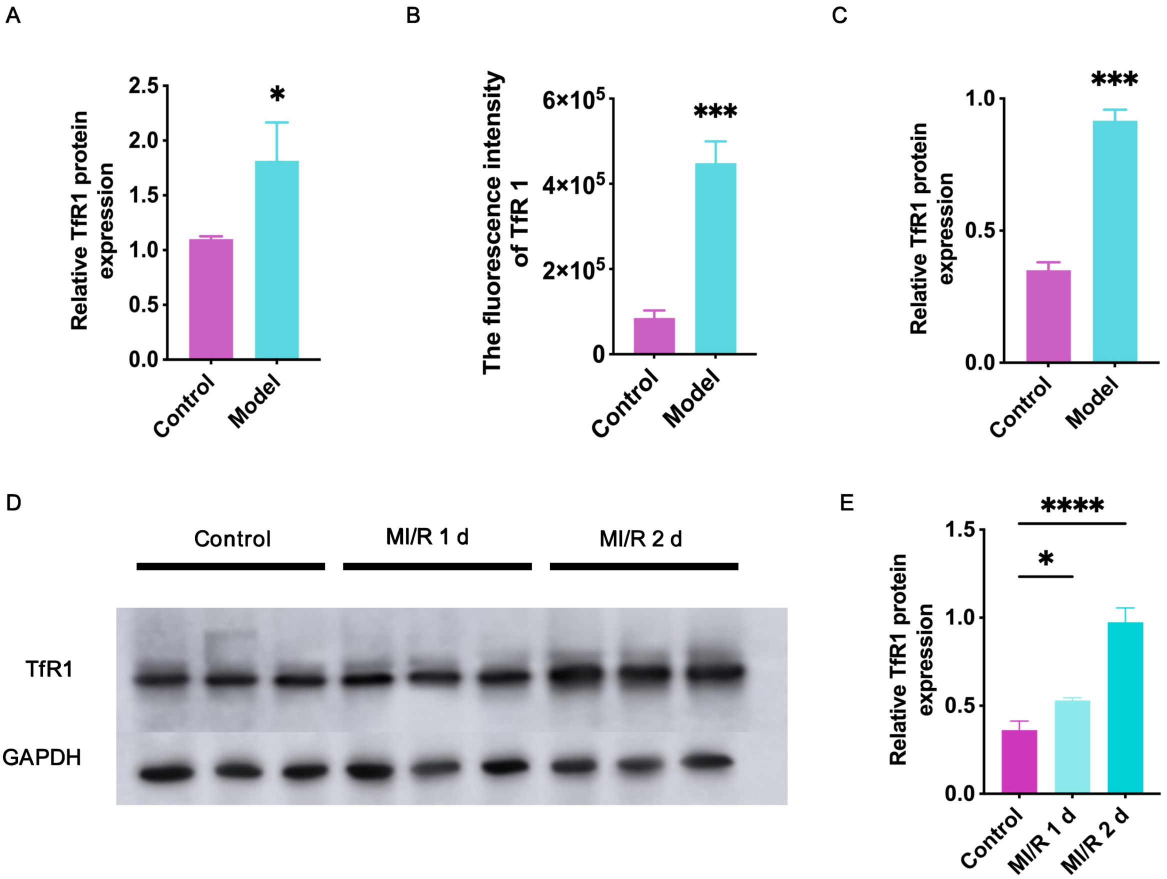
A
B
C
Relative TfR1 protein expression
2.5
2.0
1.5
1.0
0.5
0.0
*
Control
Model
The fluorescence intensity of TfR 1
6×10^5
4×10^5
2×10^5
0
***
Control
Model
Relative TfR1 protein expression
1.0
0.5
0.0
***
Control
Model
D
Control
MI/R 1 d
MI/R 2 d
TfR1
GAPDH
E
****
*
Relative TfR1 protein expression
1.5
1.0
0.5
0.0
Control
MI/R 1 d
MI/R 2 d

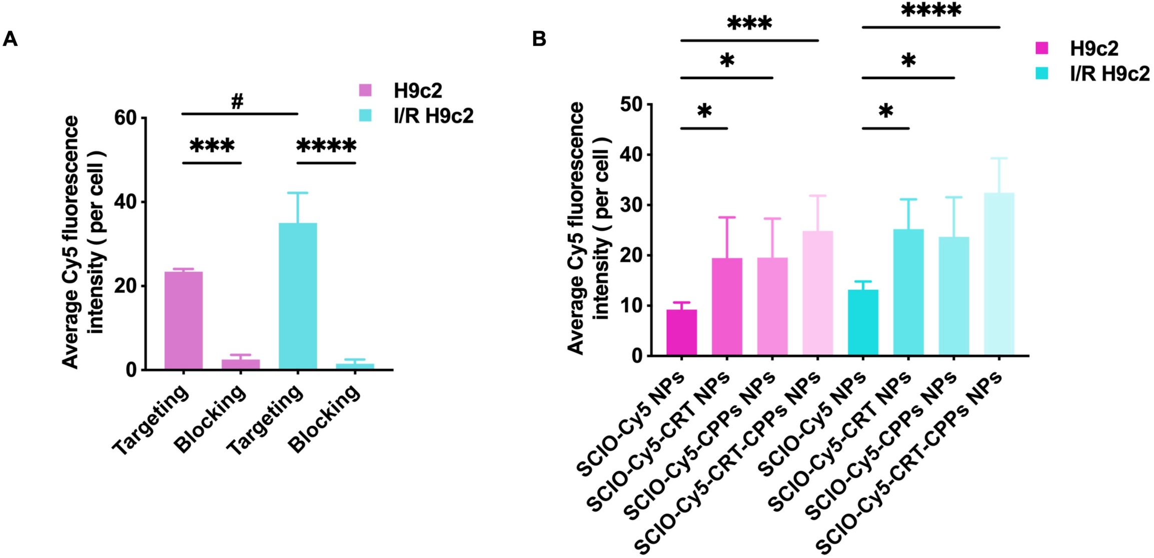
A
Average Cy5 fluorescence intensity (per cell)
60
40
20
0
#
***
****
H9c2
I/R H9c2
Targeting
Blocking
Targeting
Blocking
B
***
****
*
*
*
*
H9c2
I/R H9c2
Average Cy5 fluorescence intensity (per cell)
50
40
30
20
10
0
SCIO-Cy5 NPs
SCIO-Cy5-CRT NPs
SCIO-Cy5-CPPs NPs
SCIO-Cy5-CRT-CPPs NPs
SCIO-Cy5 NPs
SCIO-Cy5-CRT NPs
SCIO-Cy5-CPPs NPs
SCIO-Cy5-CRT-CPPs NPs

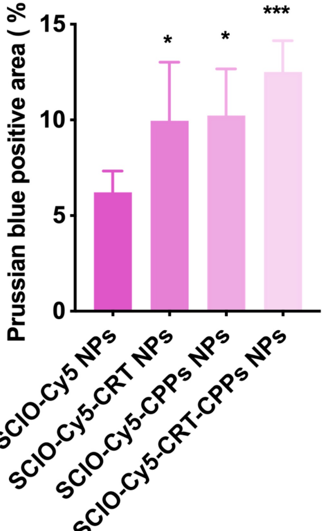

Prussian blue positive area (%)
15
10
5
0
*
*
***
SCIO-Cy5 NPs
SCIO-Cy5-CRT NPs
SCIO-Cy5-CPPs NPs
SCIO-Cy5-CRT-CPPs NPs

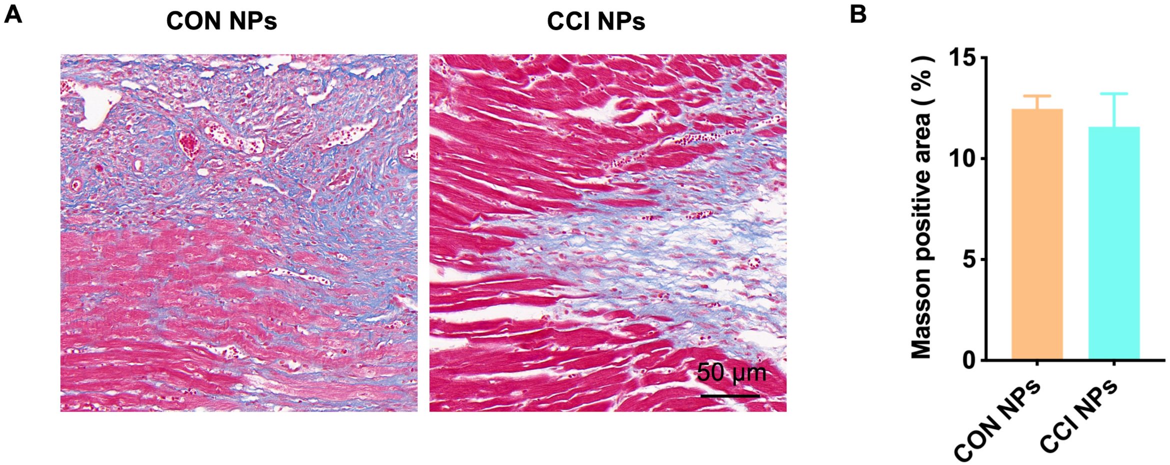
A
CON NPs
CCI NPs
50 μm
B
Masson positive area (%)
15
10
5
0
CON NPs
CCI NPs

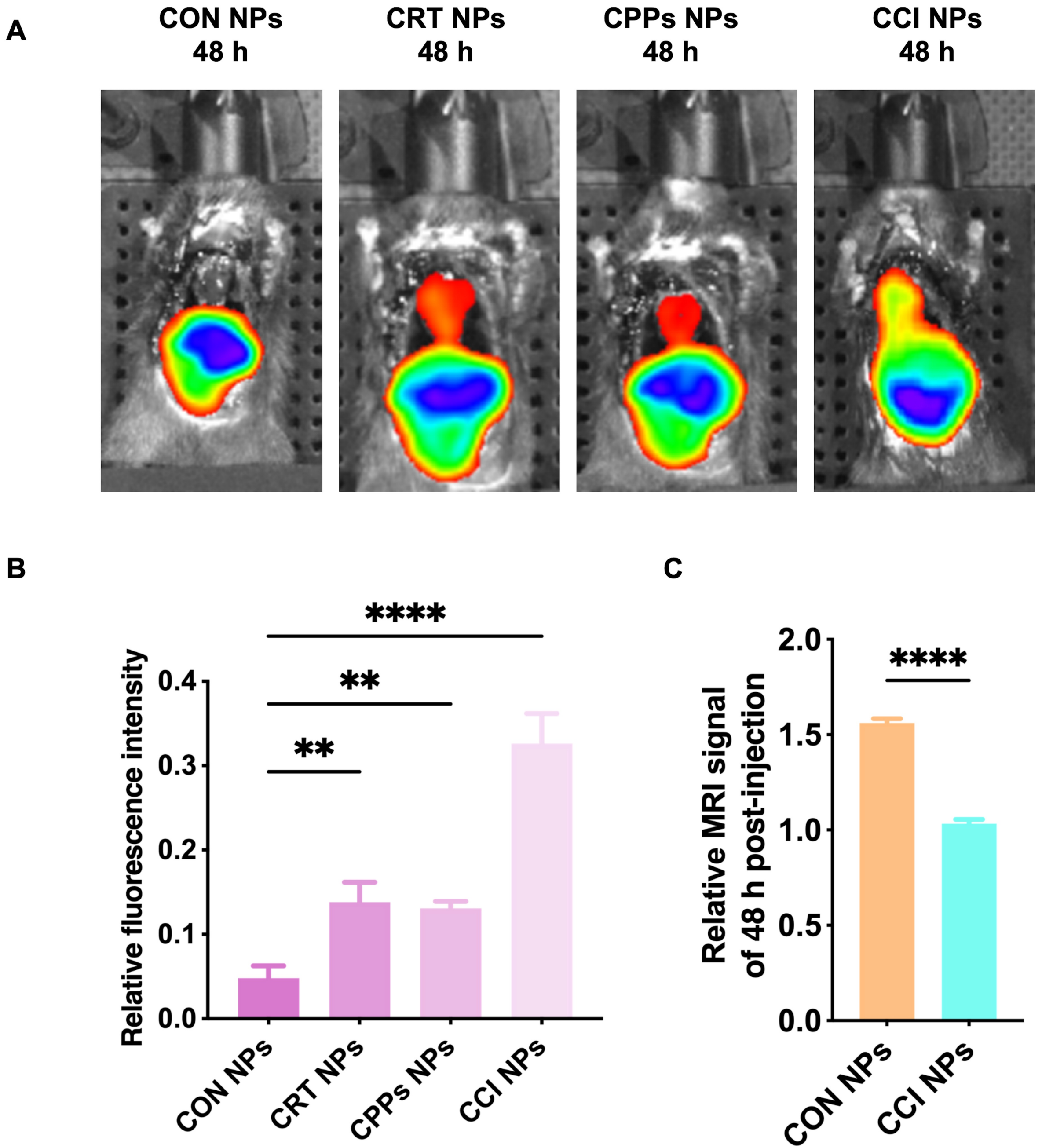
A
CON NPs
48 h
CRT NPs
48 h
CPPs NPs
48 h
CCI NPs
48 h
B
****
**
**
Relative fluorescence intensity
0.4
0.3
0.2
0.1
0.0
CON NPs
CRT NPs
CPPs NPs
CCI NPs
C
****
Relative MRI signal
of 48 h post-injection
2.0
1.5
1.0
0.5
0.0
CON NPs
CCI NPs

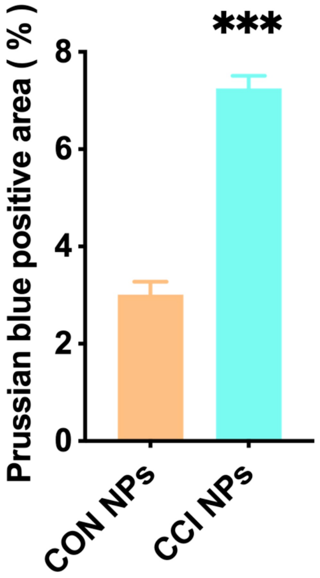

Prussian blue positive area (%)
***
8
6
4
2
0
CON NPs
CCI NPs

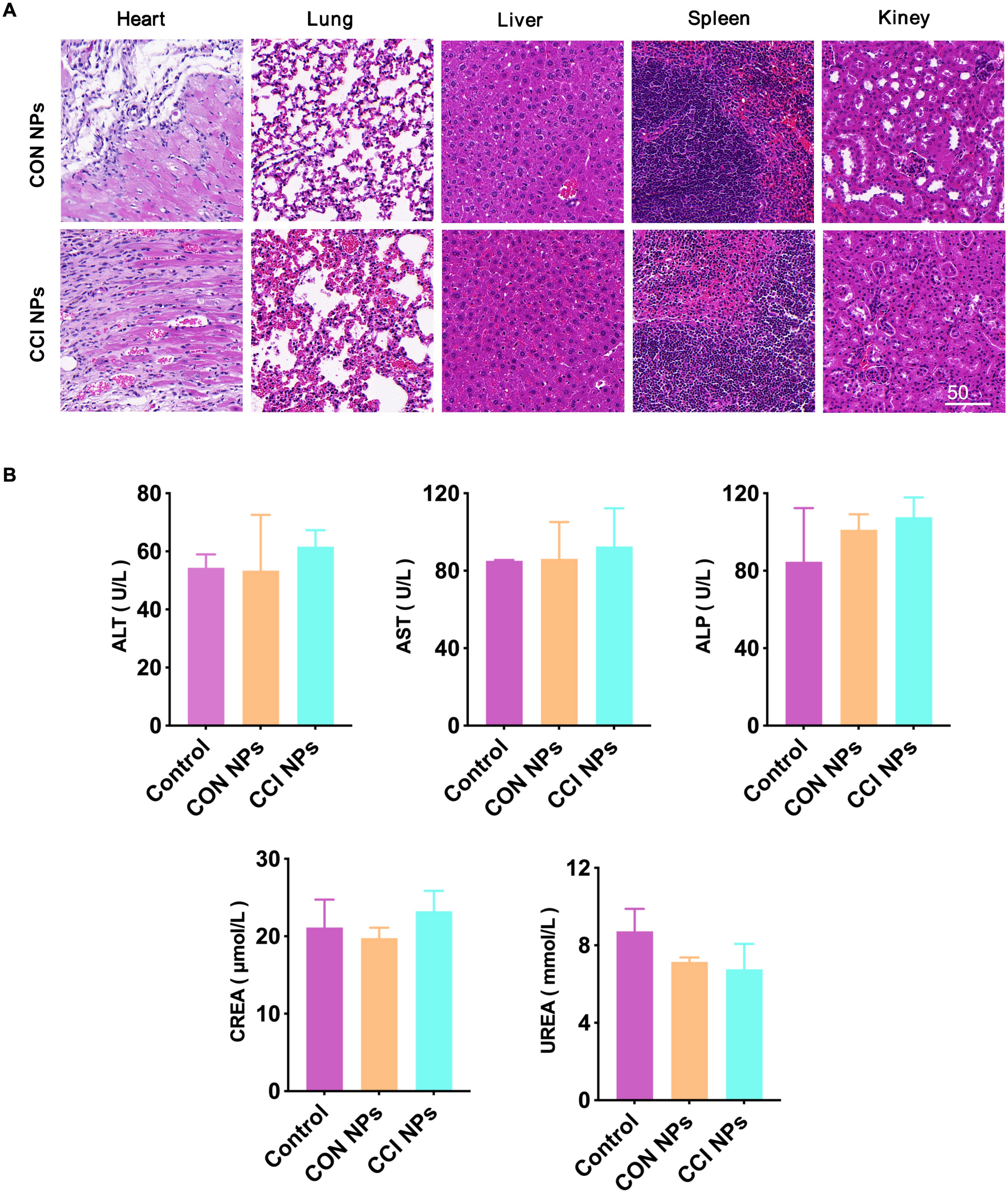
A
Heart
Lung
Liver
Spleen
Kiney
CON NPs
CCI NPs
50
B
80
60
40
20
0
ALT (U/L)
Control
CON NPs
CCI NPs
120
80
40
0
AST (U/L)
Control
CON NPs
CCI NPs
120
80
40
0
ALP (U/L)
Control
CON NPs
CCI NPs
30
20
10
0
CREA (μmol/L)
Control
CON NPs
CCI NPs
12
8
4
0
UREA (mmol/L)
Control
CON NPs
CCI NPs

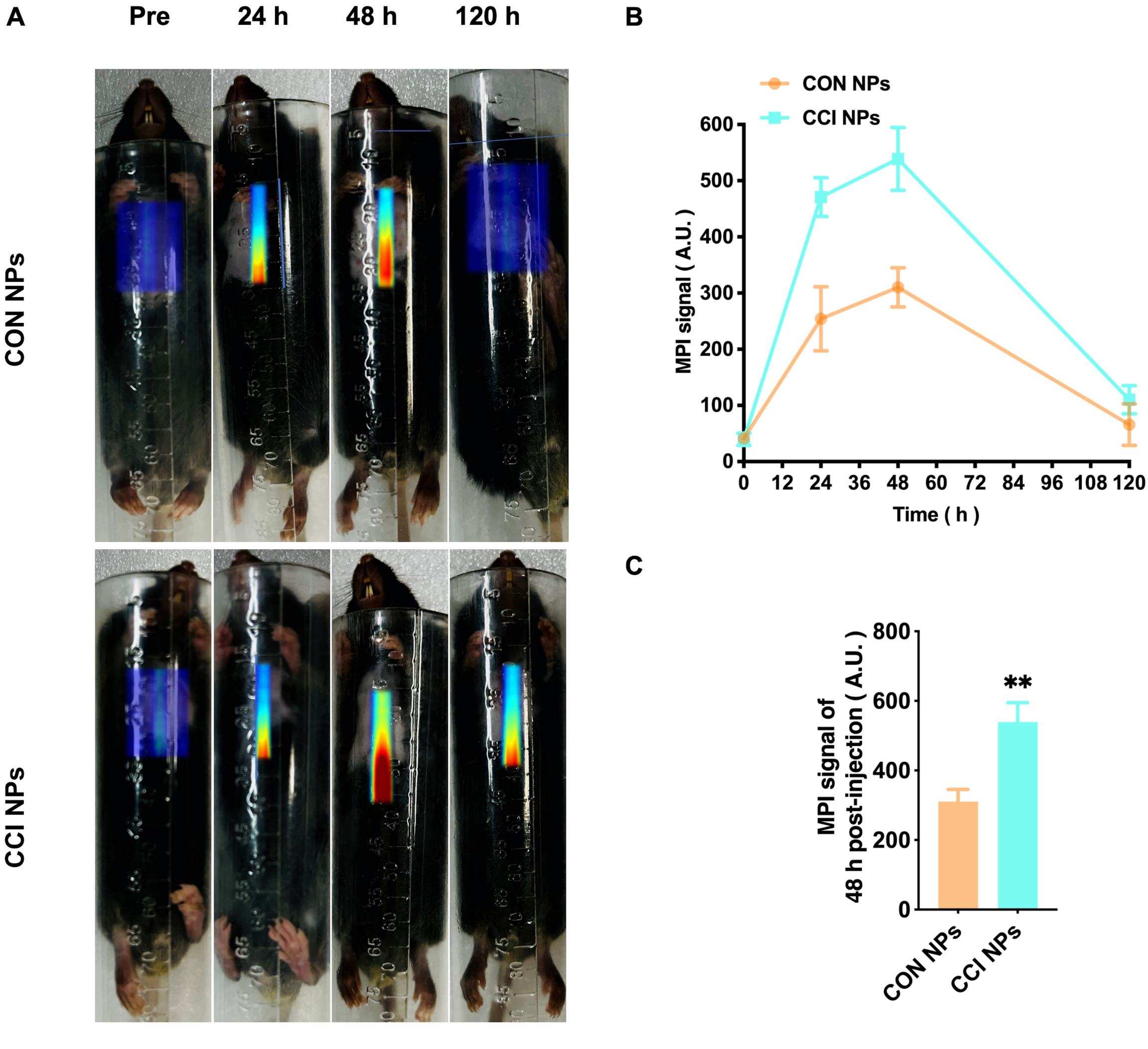
A
Pre
24 h
48 h
120 h
CON NPs
CCI NPs
B
CON NPs
CCI NPs
MPI signal (A.U.)
Time (h)
0
12
24
36
48
60
72
84
96
108
120
600
500
400
300
200
100
0
C
MPI signal of
48 h post-injection (A.U.)
800
600
400
200
0
**
CON NPs
CCI NPs
