# Supplementary material for: Quantitative visualization of myocardial ischemia-reperfusion-induced cardiac lesions via ferroptosis magnetic particle imaging
Source: Theranostics. 2024 Jan 1;14(3):1081–97. doi: 10.7150/thno.89190 (PMC10797296; doi:10.7150/thno.89190)
Supplement: Supplementary file 1 — Experimental procedures, synthetic details and characterization, additional analytical data; Figures S1-S12. 3D MPI/CT images of the MI/R mouse model incubated with CON and CCI NPs; Video S1-S2. Video of MI/R mouse model without the liver incubated with CCI NPs; Video S3. Supplementary figures and tables. [file thnov14p1081s1.zip › Supplementary Materials.docx]

Supplementary Materials for

**Quantitative visualization of myocardial ischemia–reperfusion-induced cardiac lesions via ferroptosis magnetic particle imaging**

*Establishment of DOX-induced cardiac injury Animal Model*

Seven-week-old C57BL/6N mice were purchased from Beijing Charles River Experimental Animal Technology Company, China. In the doxorubicin-induced cardiac injury mouse model, In this respect, we used the CCI and CON NPs to perform MPI imaging (Self-developed small animal Field Free Line-MPI scanner) [1] 48 h after giving DOX (10 mg/kg) intraperitoneally to healthy mice [2], and performed image reconstruction with MATLAB (Mathworks MATLAB, MA, R2022B).


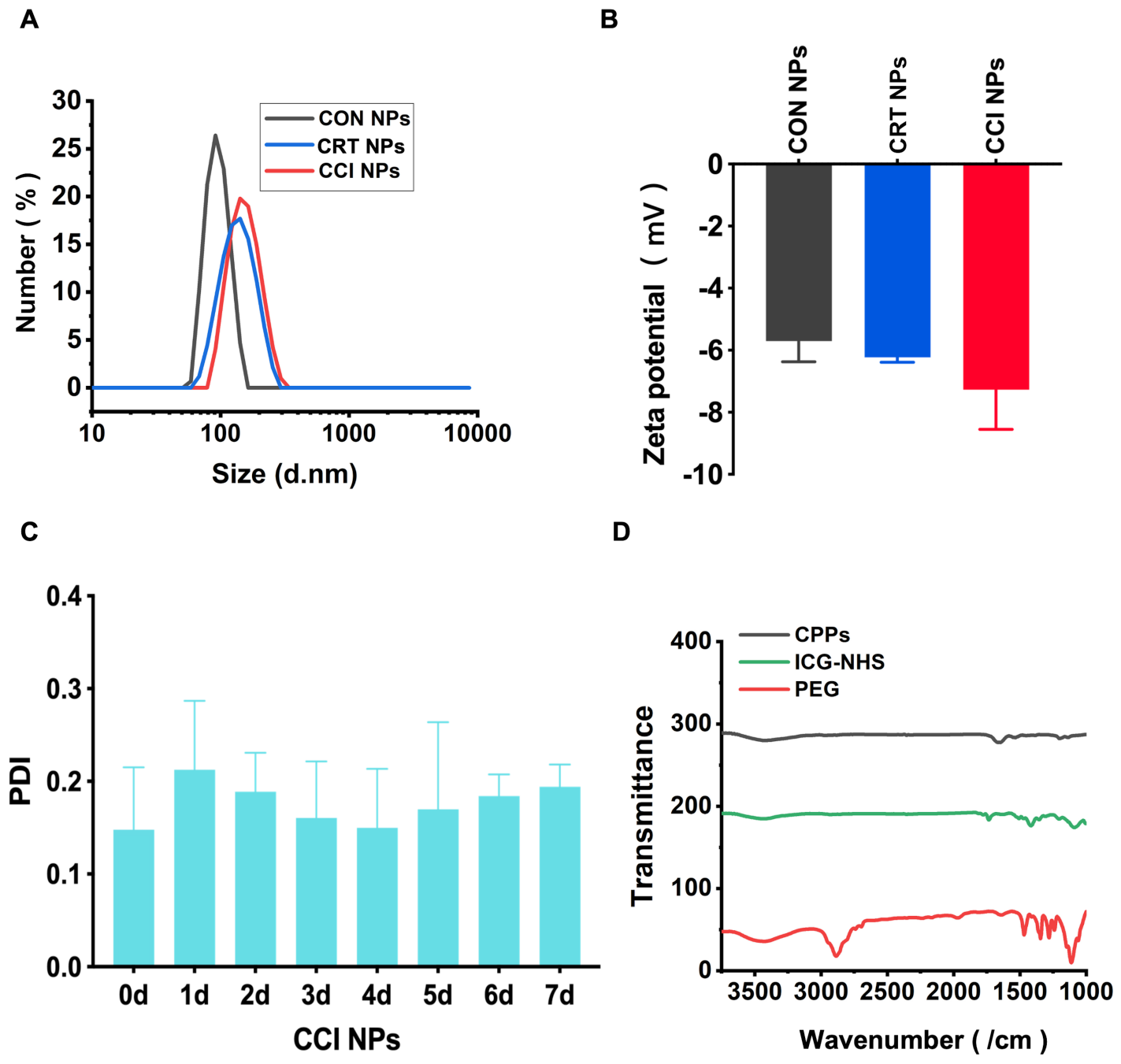


**Figure S1.** Characterization of CCI NPs. (A) Hydrated particle size of CON, CRT, and CCI NPs. (B) Zeta potential of CON, CRT, and CCI NPs (n = 3, respectively; Student’s *t test. * p <* 0.05*; ** p <* 0.01*; *** p <* 0.001*, and **** p <* 0.0001). (C) PDI of CCI NPs (n = 3, respectively; Student’s *t test. * p <* 0.05*; ** p <* 0.01*; *** p <* 0.001*, and **** p <* 0.0001). (D) FTIR spectra of CPPs, ICG-NHS, and PEG.


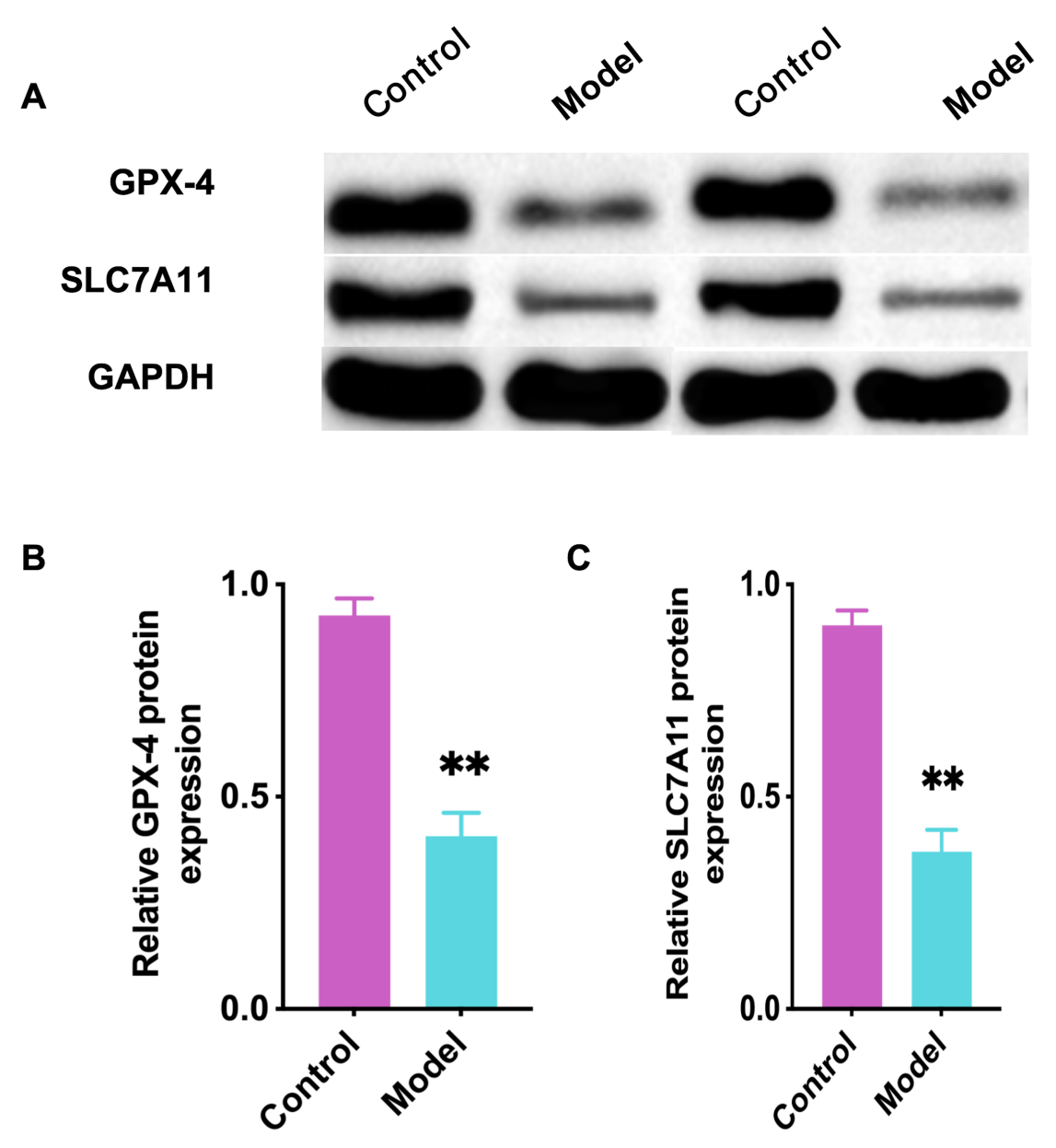


**Figure S2.** (A) The expression of GPX-4 and SLC7A11 in normal and I/R H9c2 cells (Control *vs.* Model) was detected via western blot with GAPDH used as reference (representative blots). The western blot analysis of GPX-4 (B) and SLC7A11(C) in cells, with a sample size of n = 3, respectively. (Student’s *t test. * p < 0.05; ** p < 0.01; *** p < 0.001, and **** p < 0.0001*).


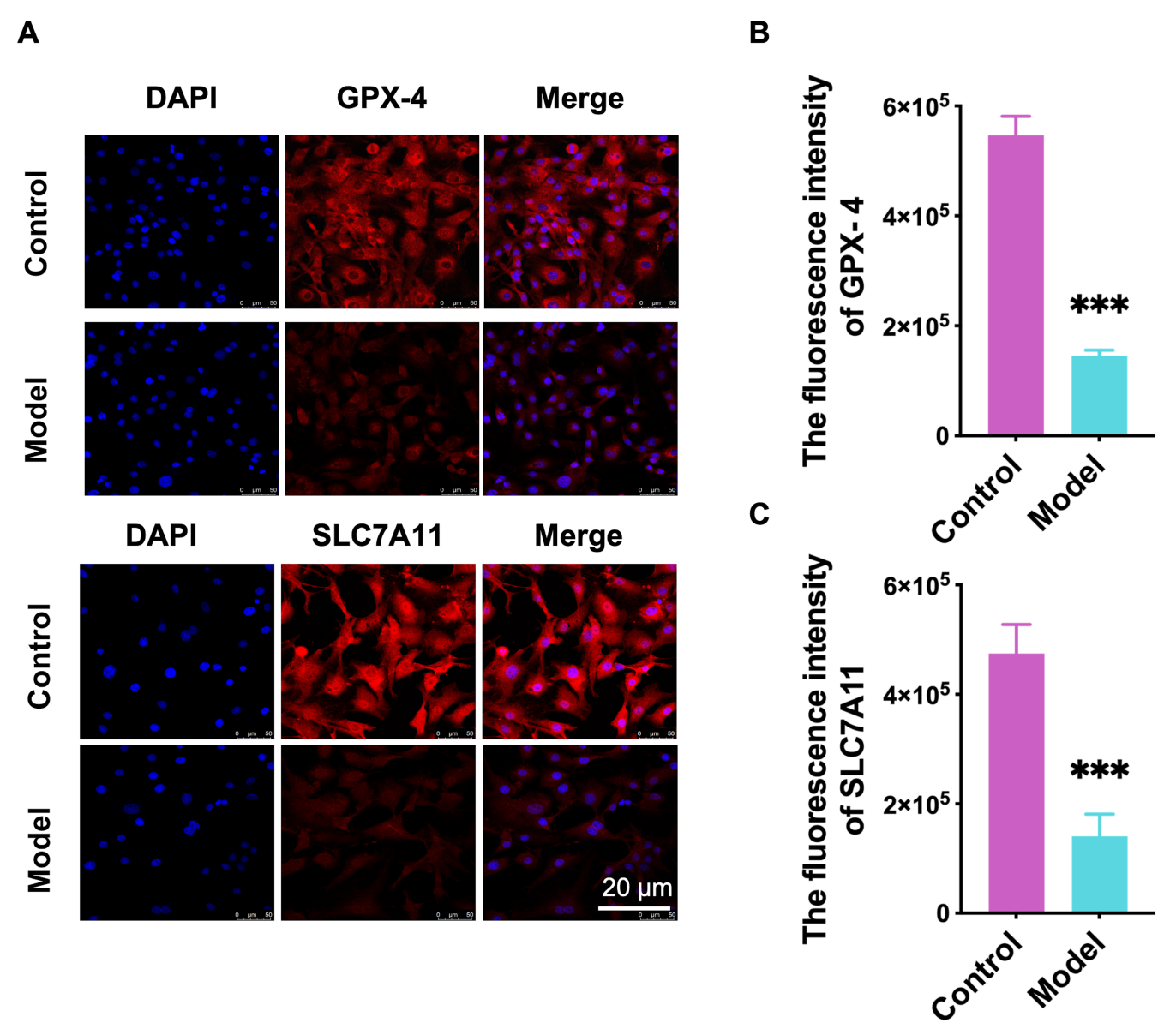


**Figure S3.** (A) The expression of GPX-4 and SLC7A11 in normal and I/R H9c2 cells (Control *vs.* Model) was detected via immunofluorescent staining (representative images). The immunofluorescent staining analysis of GPX-4 (B) and SLC7A11(C) in cells, with a sample size of n = 6, respectively. (Student’s *t test. * p < 0.05; ** p < 0.01; *** p < 0.001, and **** p < 0.0001*).


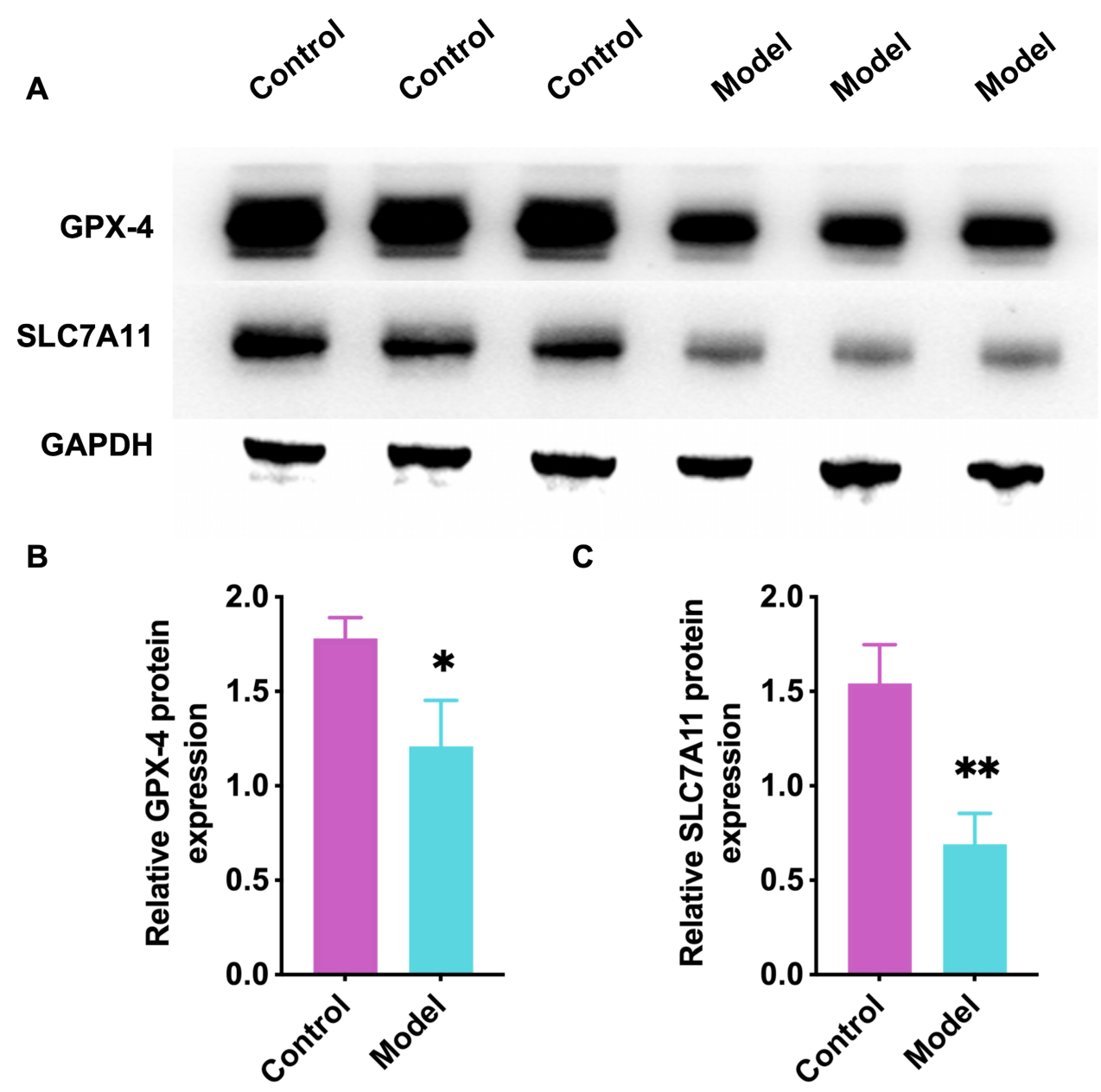


**Figure S4.** (A) The expression of GPX-4 and SLC7A11 in normal mouse and MI/R mouse model (Control *vs.* Model) was detected via western blot (representative blots). The western blot analysis of GPX-4 (B) and SLC7A11(C) in mouse. (n = 3, respectively; Student’s *t test. * p < 0.05; ** p < 0.01; *** p < 0.001, and **** p < 0.0001*).


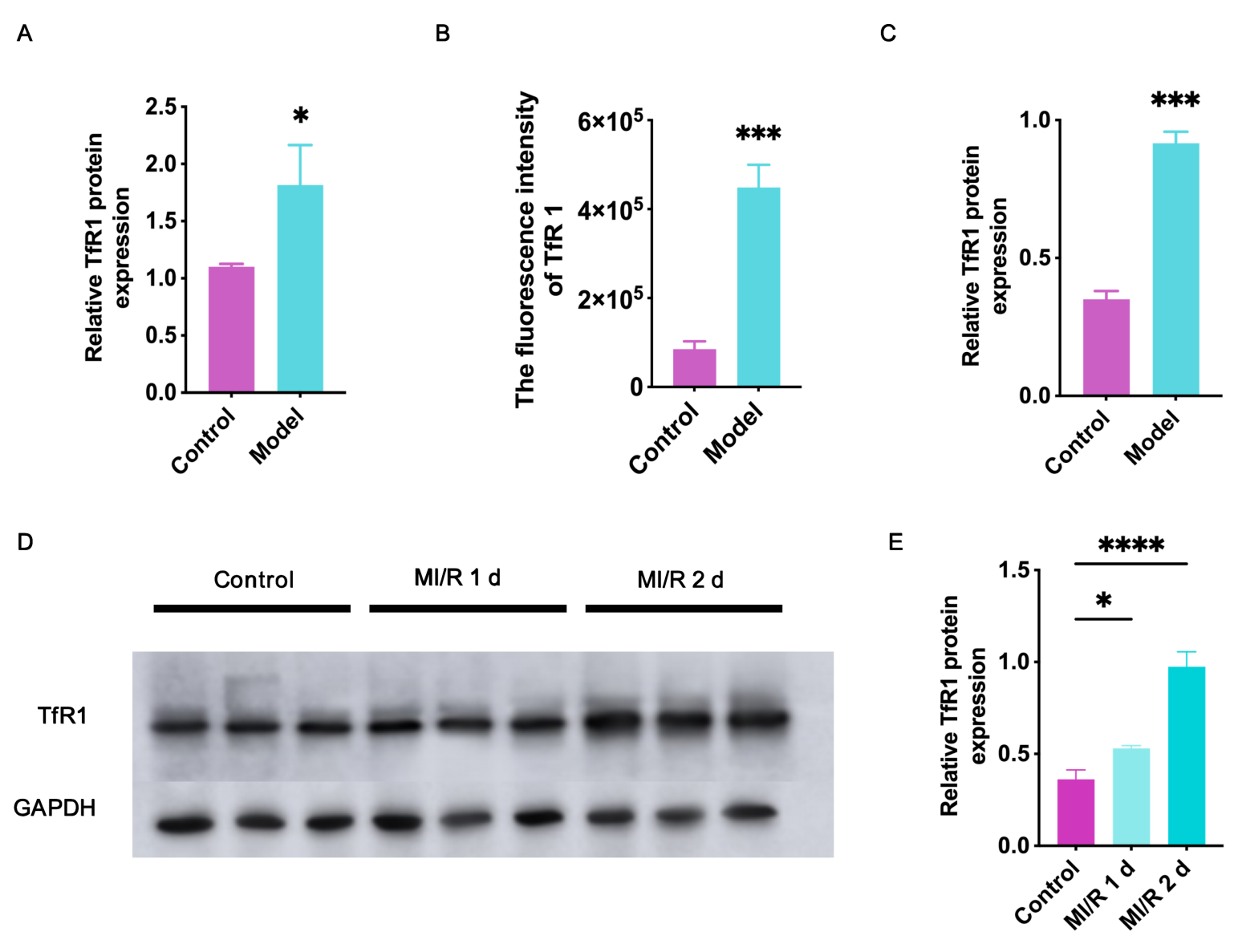


**Figure S5.** (A)The analysis of western blot in Figure 2B. The normal and I/R H9c2 cells (n = 3; Control *vs.* Model). (B) The fluorescent intensity of the fluorescent images in Figure 2C (n = 6). The normal and I/R H9c2 cells (Control *vs.* Model). (C) The relative TfR1 protein expression of western blot in Figure 2E. The normal mouse and MI/R mouse model (Control *vs.* Model). (n = 3, respectively; Student’s *t test. * p < 0.05; ** p < 0.01; *** p < 0.001, **** p < 0.0001*). (D) The expression of TfR1 in MI/R mouse model of different day (Control *vs.* MI/R 1 d *vs.* MI/R 2 d). (E) The western blot analysis of TfR1 in MI/R mouse model of different day. (n = 3, respectively; Student’s t test. ** p < 0.05; ** p < 0.01; *** p < 0.001, and **** p < 0.0001*).


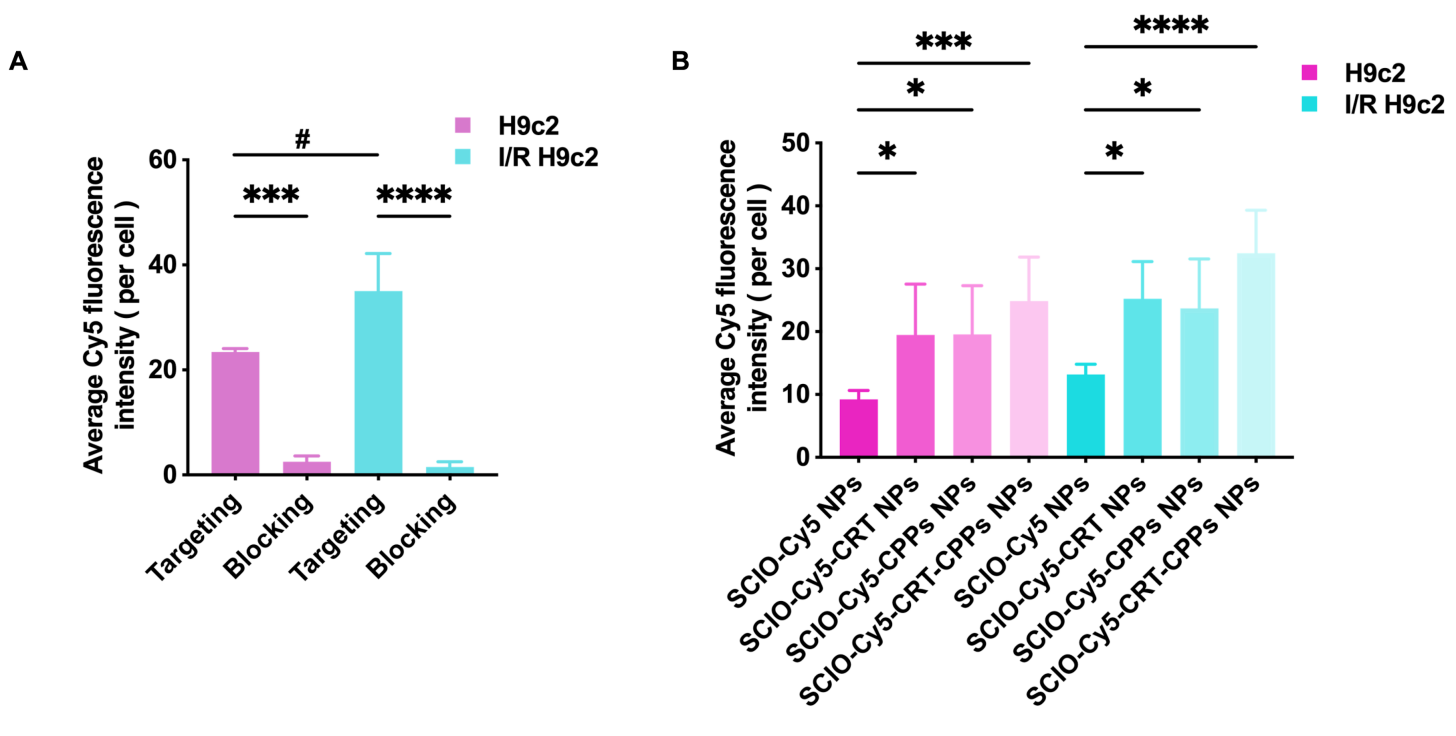


**Figure S6.** (A) The fluorescence intensities of fluorescent images in Figure 3A. (n = 6; *one-way ANOVA, *p < 0.05* considered significant;** p < 0.05; ** p < 0.01; *** p < 0.001, **** p < 0.0001; ^#^p < 0.05* considered significant of comparison between groups). (B) The analysis of relative fluorescence intensities of fluorescent images of Figure 3D. (n = 6, respectively; *one-way ANOVA, *p < 0.05* considered significant;** p < 0.05; ** p < 0.01; *** p < 0.001, and **** p < 0.0001*).


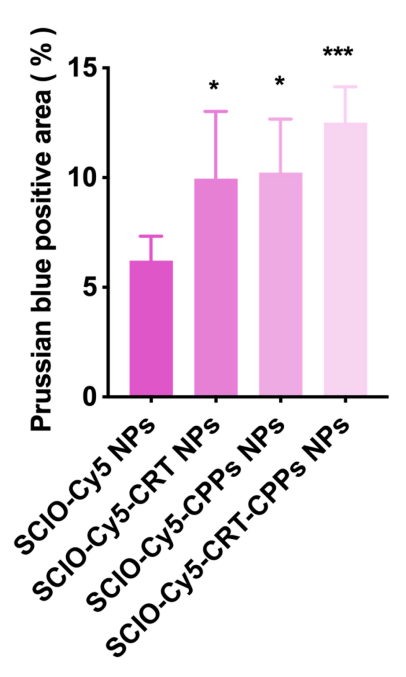


**Figure S7.** The analysis of Prussian blue-positive areas in normal H9c2 cells co-cultured with SCIO-Cy5/SCIO-Cy5-CRT/SCIO-Cy5-CPPs/SCIO-Cy5-CRT-CPPs NPs in Figure 3F. (n = 6, respectively; *one-way ANOVA, *p < 0.05* considered significant;** p < 0.05; ** p < 0.01; *** p < 0.001, and **** p < 0.0001*).


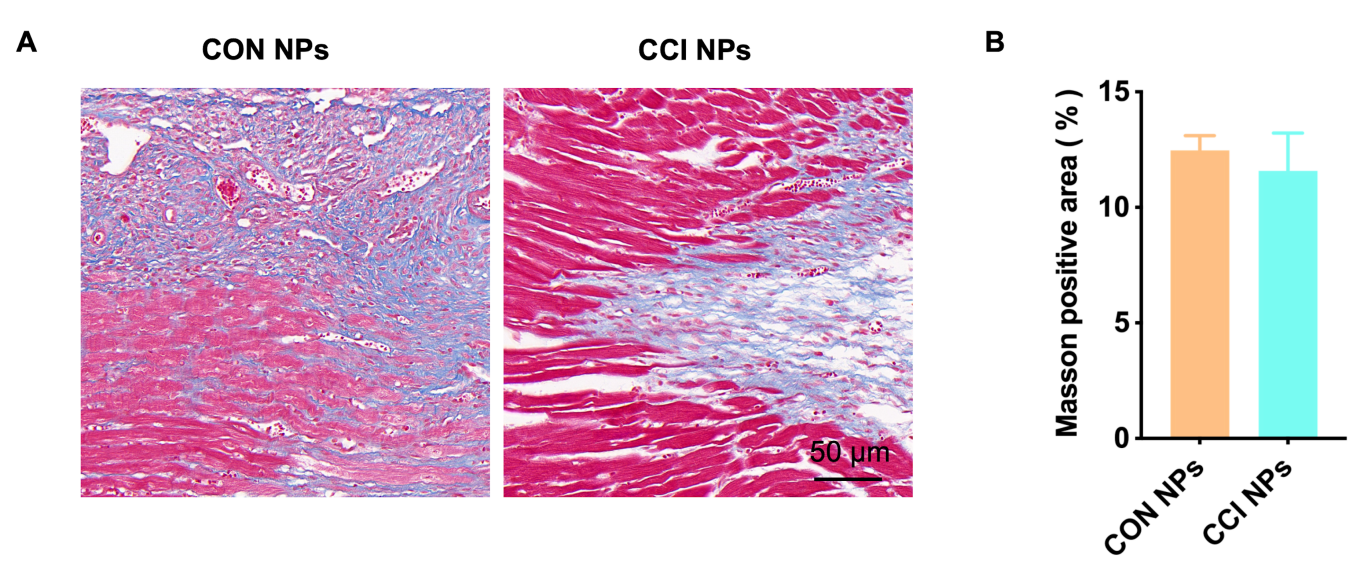


**Figure S8.** (A) The Masson staining images of resected MI/R hearts in MI/R mouse model. (CON NPs *vs.* CCI NPs). (B) The analysis of Masson positive area. (n = 3, respectively; Student’s *t test. *p < 0.05 considered significant;* p < 0.05; ** p < 0.01; *** p < 0.001, and **** p < 0.0001*).


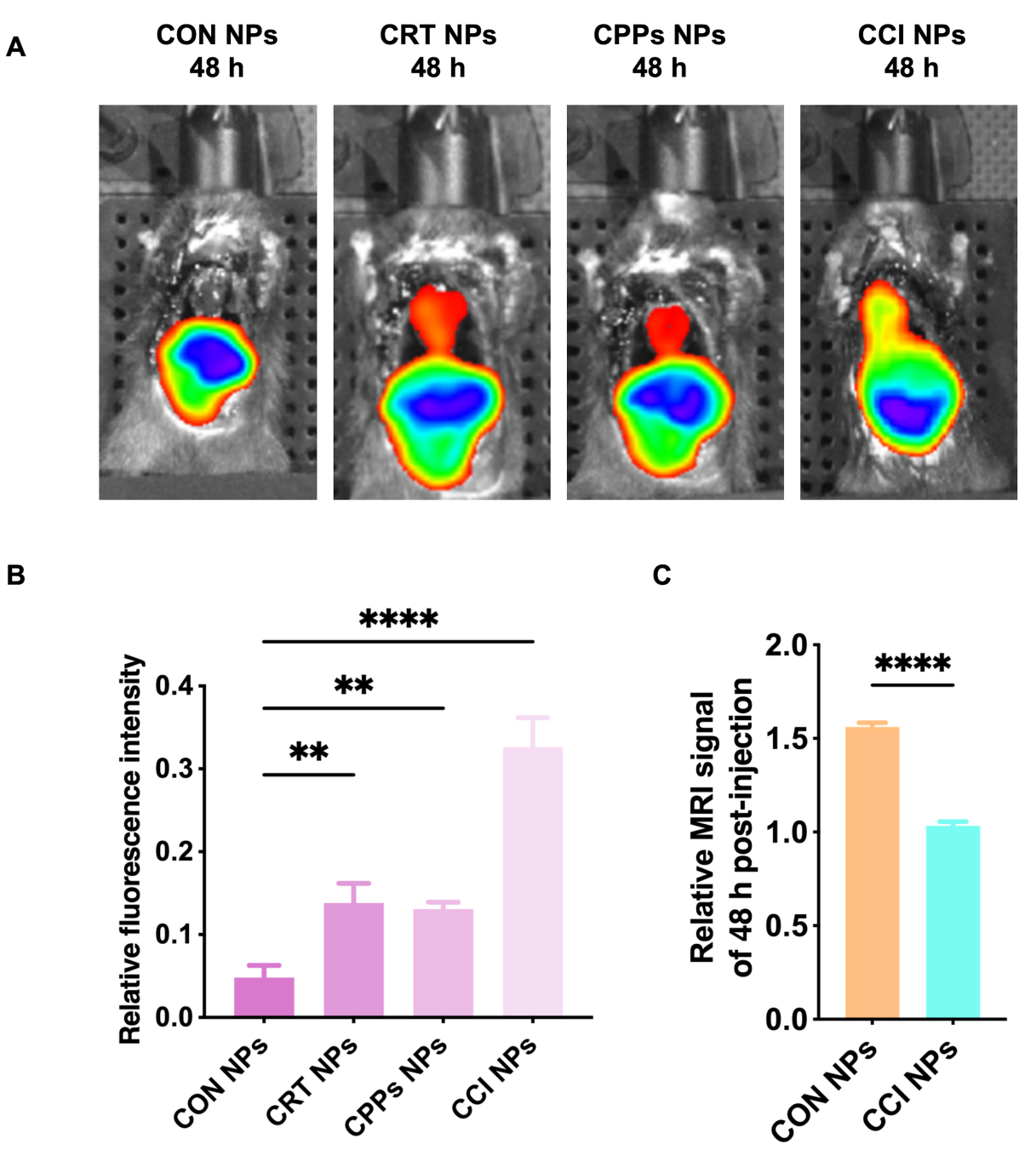


**Figure S9**. (A) The NIR fluorescent images of the MI/R mouse model at 48 h post-injection *ex vivo* from different groups (CON NPs *vs.* CRT NPs *vs.* CPPs NPs *vs.* CCI NPs; n = 3). (B) Quantitative comparison of relative fluorescence intensities of CON, CRT, CPPs, and CCI NPs (n = 3). (C) The 48 h post-injection relative MRI signal was compared for different groups (CON NPs *vs.* CCI NPs; n = 3; Student’s *t-test. *p < 0.05; **p < 0.01; ***p < 0.001; and ****p < 0.0001*)


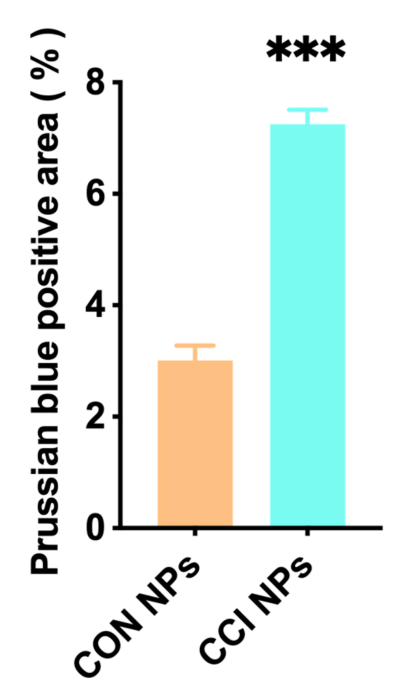


**Figure S10.** The analysis of Prussian blue positive area of resected MI/R hearts in Figure 6C. (CON NPs *vs.* CCI NPs). (n = 3, respectively; Student’s *t test. *p < 0.05 considered significant;* p < 0.05; ** p < 0.01; *** p < 0.001, and **** p < 0.0001*).


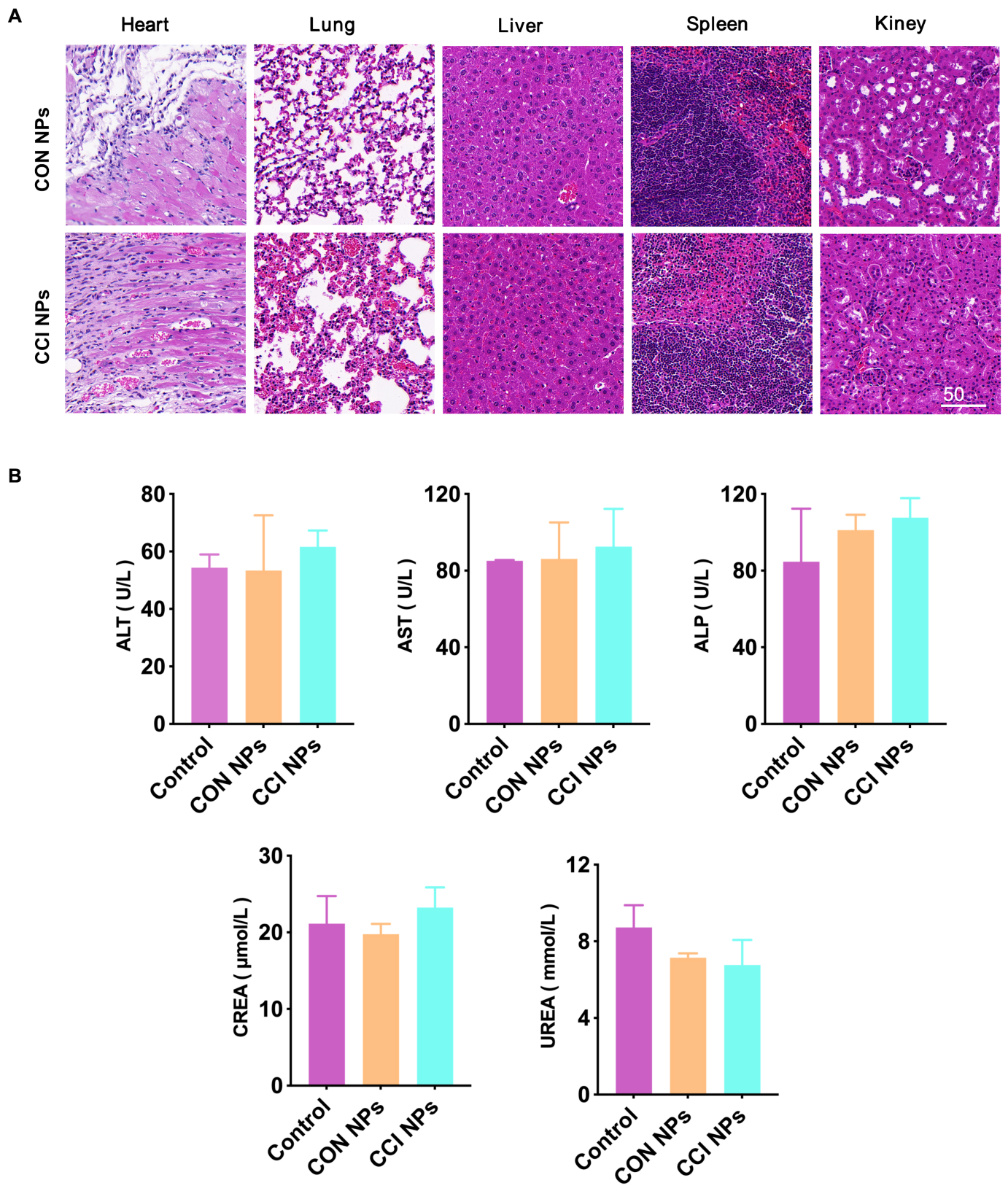


**Figure S11.** Evaluation of systemic toxicity after imaging *in vivo*. (A) H&E staining of major organs in MI/R mouse model (CON NPs and CCI NPs, respectively). (Scale bar: 50 μm). (B) Liver and kidney function was evaluated through the measurement of ALT, AST, ALP, CREA, and UREA levels in normal mouse (Control), and MI/R mouse models (CON and CCI NPs). (n = 3, respectively; *one-way ANOVA, *p < 0.05 considered significant;* p < 0.05; ** p < 0.01; *** p < 0.001, and **** p < 0.0001*).


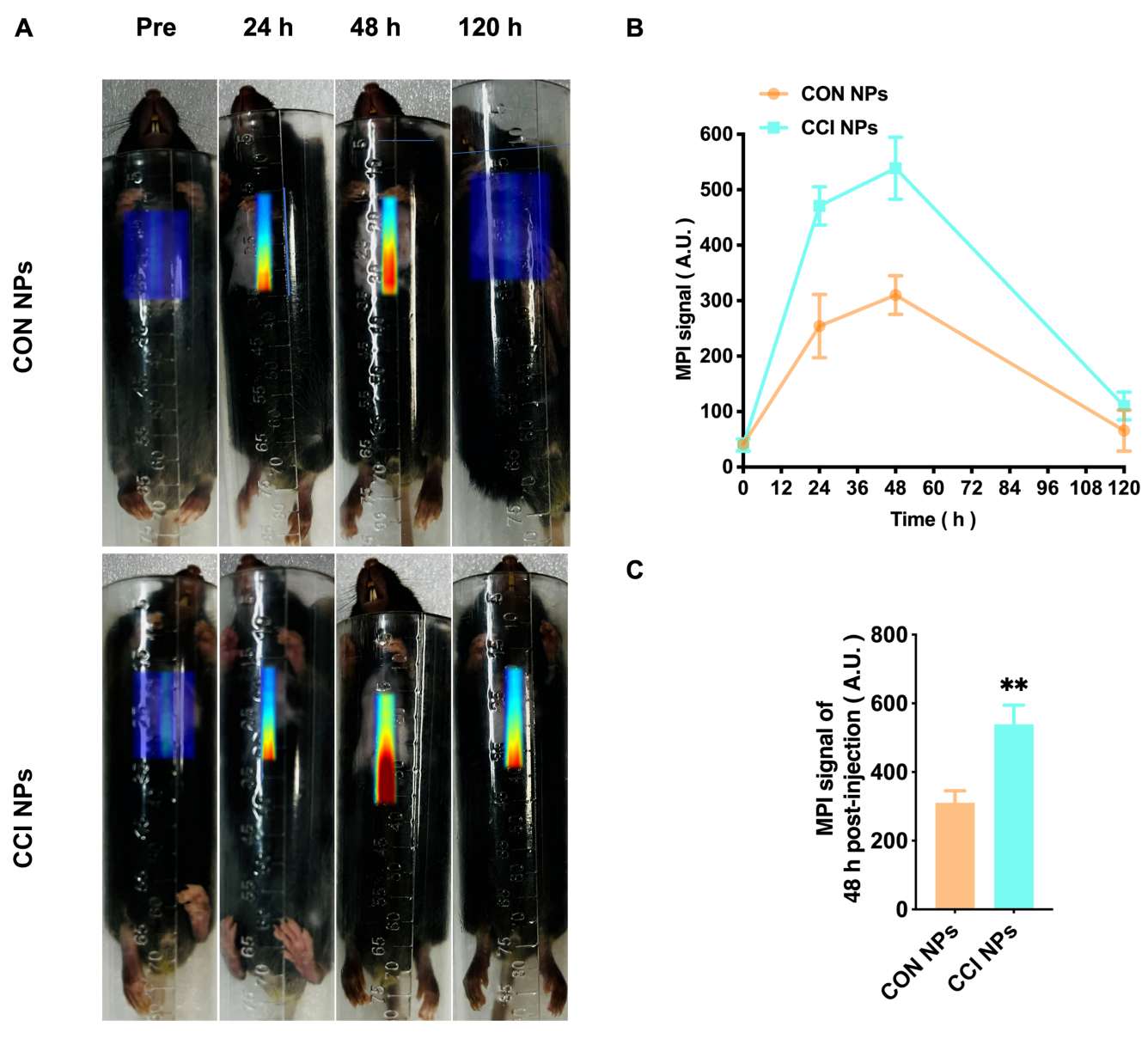


**Figure S12.** (A) *In vivo* DOX-induced cardiac injury mouse model from different groups (CON NPs *vs.* CCI NPs) at different time points (0, 24, 48, and 120 h) of MPI images. (B) Quantitative comparison of MPI signals of NPs (n = 3). (D) The MPI signal in cardiac of 48 h post-injection was compared for different groups (CON NPs *vs.* CCI NPs; n = 3; Student’s *t-test. *p < 0.05; **p < 0.01; ***p < 0.001; and ****p < 0.0001*).

**Video S1.**

This movie presents a 3D MPI/CT signal distribution of CON NPs in a living MI/R mouse model, captured 48 h after injection.

**Video S2.**

This movie presents a 3D MPI/CT signal distribution of CCI NPs in a living MI/R mouse model, captured 48 h after injection.

**Video S3.**

This movie presents a 3D MPI/CT signal distribution of CCI NPs in MI/R mouse model without the liver at 48 h post-injection.

**References**

1. Vogel P, Rückert MA, Friedrich B, Tietze R, Lyer S, et al. Critical Offset Magnetic PArticle SpectroScopy for rapid and highly sensitive medical point-of-care diagnostics. Nat Commun. 2022; 13:7230.
2. Zeng F, Nijiati S, Liu Y, Yang Q, Liu X, et al. Ferroptosis MRI for early detection of anticancer drug-induced acute cardiac/kidney injuries. Sci Adv. 2023; 9:eadd8539.
