# Supplementary figures and images for: Quantitative visualization of myocardial ischemia-reperfusion-induced cardiac lesions via ferroptosis magnetic particle imaging
Source: Theranostics. 2024 Jan 1;14(3):1081–97. doi: 10.7150/thno.89190 (PMC10797296; doi:10.7150/thno.89190)

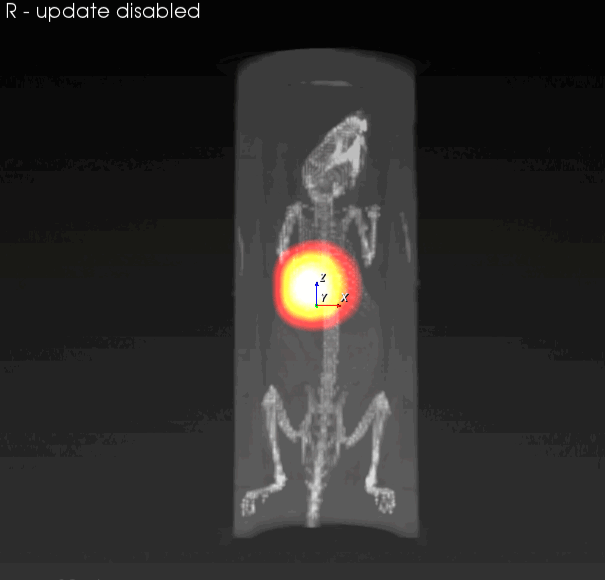

Supplement: Supplementary file 1 — Experimental procedures, synthetic details and characterization, additional analytical data; Figures S1-S12. 3D MPI/CT images of the MI/R mouse model incubated with CON and CCI NPs; Video S1-S2. Video of MI/R mouse model without the liver incubated with CCI NPs; Video S3. Supplementary figures and tables. [file thnov14p1081s1.zip › Video S1.GIF]

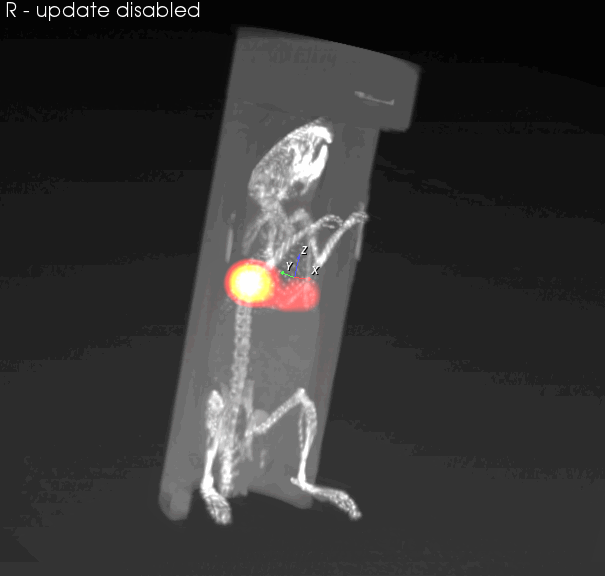

Supplement: Supplementary file 1 — Experimental procedures, synthetic details and characterization, additional analytical data; Figures S1-S12. 3D MPI/CT images of the MI/R mouse model incubated with CON and CCI NPs; Video S1-S2. Video of MI/R mouse model without the liver incubated with CCI NPs; Video S3. Supplementary figures and tables. [file thnov14p1081s1.zip › Video S2.GIF]

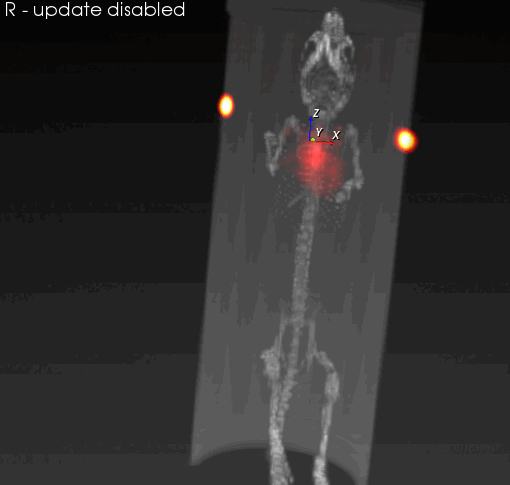

Supplement: Supplementary file 1 — Experimental procedures, synthetic details and characterization, additional analytical data; Figures S1-S12. 3D MPI/CT images of the MI/R mouse model incubated with CON and CCI NPs; Video S1-S2. Video of MI/R mouse model without the liver incubated with CCI NPs; Video S3. Supplementary figures and tables. [file thnov14p1081s1.zip › Video S3.GIF]
